# The Environmental Spread of *Aspergillus terreus* in Tyrol, Austria

**DOI:** 10.3390/microorganisms9030539

**Published:** 2021-03-05

**Authors:** Anna-Maria Dietl, Roya Vahedi-Shahandashti, Carmen Kandelbauer, Bart Kraak, Michaela Lackner, Jos Houbraken, Cornelia Lass-Flörl

**Affiliations:** 1 Institute of Hygiene & Medical Microbiology, Medical University of Innsbruck, 6020 Innsbruck, Austria; anna-maria.dietl@i-med.ac.at (A.-M.D.); roya.vahedi@i-med.ac.at (R.V.-S.); carmen.kandelbauer@i-med.ac.at (C.K.); michaela.lackner@i-med.ac.at (M.L.); 2 Westerdijk Fungal Biodiversity Institute, 3584 CT Utrecht, The Netherlands; b.kraak@wi.knaw.nl (B.K.); j.houbraken@wi.knaw.nl (J.H.)

**Keywords:** *Aspergillus terreus*, environmental distribution, epidemiology, antifungal susceptibility testing, environmental exposure, airborne pathogen, soil, genotyping, short tandem repeat, azole, amphotericin B, resistance

## Abstract

Fungal infections due to *Aspergillus* species have become a major cause of morbidity and mortality among immunocompromised patients. At the Medical University of Innsbruck, *A. terreus* and related species are the second most common causative agents of aspergillosis. In this one-year study we collected environmental samples to investigate (i) the environmental distribution, (ii) the ecological niche of *A. terreus* in Tyrol, (iii) the genetic relatedness of environmental and clinical isolates and the correlation between those two groups of isolates, and (iv) the antifungal susceptibility patterns. *A. terreus* was present in 5.4% of 3845 environmental samples, with a significantly higher frequency during winter (6.8%) than summer (3.9%). An increased *A. terreus* abundance in Tyrol’s Eastern part was detected which is in agreement with the proof of clinical cases. In total, 92% of environmental and 98% of clinical *A. terreus* isolates were amphotericin B resistant; 22.6% and 9.8% were resistant against posaconazole. Overall, 3.9% of clinical isolates were resistant against voriconazole. Short tandem repeat analysis identified three major genotypes persisting in Tyrol. Soil from agricultural cornfields seems to be an important source; the environmental frequency of *A. terreus* correlates with the high incidence of *A. terreus* infections in certain geographical areas.

## 1. Introduction

*Aspergillus* species are the most prevalent airborne fungal pathogens associated with significant morbidity and mortality mainly in immunocompromised patients [1]. The primary human pathogenic *Aspergillus* species belong to *Aspergillus* section *Fumigati*, followed by members of sections *Flavi*, *Nigri,* and *Terrei* [2,3,4,5,6,7,8]. *Aspergillus terreus*, classified in section *Terrei*, is the most common species of the section and found worldwide in various environmental habitats [9]. This species and closely related taxa cause both systemic and superficial infections, acute and chronic diseases, and are associated with hypersensitivity [4,5,10,11,12,13,14]. *A. terreus* is the third most common filamentous fungus from respiratory secretions of cystic fibrosis patients [15,16,17].

*Aspergillus* section *Terrei* includes 17 accepted species distributed over three series (ser. *Ambigui*, *Nivei* and *Terrei*) [18]. Within the section, *A. terreus*, *A. alabamensis*, *A. citrinoterreus*, *A. floccosus*, *A. hortae*, *A. neoafricanus* (all ser. *Terrei*) and *A. niveus* (ser. *Nivei*) were found to be of clinical relevance [4,9,19,20,21]. *A. terreus* and other section *Terrei* species hold a prominent position due to intrinsic resistance against amphotericin B (AMB) [4,22,23]. Reduced azole-susceptibility was recently reported in *A. terreus* [24,25]. A specific hallmark of this fungal pathogen is dissemination, which possibly is related to the production of accessory conidia [26,27,28].

A high infection prevalence was reported from Houston/USA, Birmingham/USA, and Innsbruck/Austria [10,12,29]. At the University Hospital of Innsbruck, infections caused by *A. terreus* have been recognized since 1994 [12], and these infections were generally associated with in-hospital plants and construction activities [30,31]. The introduction of laminar airflow units for patients at risk in the early 2000s significantly decreased invasive aspergillosis cases [12,32,33]. Presently, *A. terreus* invasive infections occur at a lower frequency; however, fungal colonization remains very common. A recent study identified three major *A. terreus* endemic genotypes being responsible for the majority of invasive infections [34]. The ecological niche and environmental distribution of *A. terreus* in the Tyrolian terrain are unknown. In this one year-study study (May 2019–April 2020), we collected environmental samples to investigate (i) the environmental distribution, (ii) the ecological niche of *A. terreus* in Tyrol, (iii) the genetic relatedness of environmental and clinical isolates and the correlation between those two groups of isolates, and (iv) the antifungal susceptibility patterns.

## 2. Materials and Methods

### 2.1. Environmental Samples

Soil samples from humus, potting soil, agricultural fields, fertile meadows, grassland, parklands, forests and alpine ecosystems, obtained from the topsoil layer were collected. Then, 2 g of each sample, in biological duplicates, was suspended in 5 mL spore buffer (0.2 M NaCl with 1% Tween20). Then, 100 µL of the supernatant was plated on Sabouraud dextrose agar (SDA). Air sampling was performed with an air ampler (Biotest Hycon RCS High Flow). Samples of 200 L were collected indoor and outdoor, respectively and measurements were taken about 100 cm above ground. Water samples derived from lakes, rivers, snow, and surface water, respectively. Then, 100 µL of the fluid was directly plated on SDA. Living and dead plant material were mainly collected from flowers, leaves, fruits, vegetables, grass, hay, moss, cones, or tree barks. Samples of decaying material were obtained from compost, organic waste, or rotten wood. Dust specimens were collected indoor and outdoor, and faces from birds, pets, and grazing animals. These probes were suspended in 5 mL spore buffer and plated on SDA (supplemented with 50 mg/L chloramphenicol) and incubated 37 °C for 3–4 days.

### 2.2. Clinical A. terreus

Parallel to the environmental study all clinical *A. terreus* isolates, which were detected from routine medical specimens at the Institute of Hygiene and Medical Microbiology of the Medical University of Innsbruck, were collected and further analyzed within this project; isolates were stored in 10% glycerol broth at −20 °C.

### 2.3. Species Identification of Environmental and Clinical A. terreus

Environmental and clinical isolates morphologically resembling *A. terreus* were selected and isolated and subsequently identified to species level by sequencing a part of the calmodulin (*CaM*) gene. Partial calmodulin gene sequences were generated after DNA extraction from 3 days old cultures grown on a malt extract agar (MEA) plate at 25 °C in darkness using the Qiagen DNeasy Ultraclean™ Microbial DNA Isolation Kit and amplification and sequencing using the primer pair CMD5 (CCGAGTACAAGGARGCCTTC) and CMD6 (CCGATRGAGGTCATRACGTGG) [35]. The PCR fragments were sequenced in both directions using the ABI Prism^®^ Big DyeTM Terminator v. 3.0 Ready Reaction Cycle sequencing Kit. Samples were analyzed on an ABI PRISM 3700 Genetic Analyzer and contigs were assembled using the forward and reverse sequences with the program SeqMan from the LaserGene (DNAStar Inc., Madison, WI, USA) package. The sequences were compared in a validated in-house database of the Westerdijk Fungal Biodiversity Centre, Utrecht, The Netherlands [18]. Generated sequences were deposited in GenBank (GenBank accession numbers: BankIt2432916: MW670911–MW671051).

### 2.4. Meteorological and Geographical Data

Meteorological data on temperature, air humidity, and wind velocity were noted during sampling. Sea levels and GFP coordinates from the site of collection were obtained from Google Maps 2020 (https://www.google.com/maps, accessed on 1 March 2021). Coordinates were entered into a geographical map using QGIS 3.12 (qgis.org, accessed on 1 March 2021), an open-source desktop GIS (Geographic Information System). The map of Tyrol was obtained from the Tiroler Rauminformationssystem (https://gis.tirol.gv.at, accessed on 1 March 2021). 

### 2.5. Antifungal Susceptibility Testing

Antifungal susceptibility testing (AFST) of *A. terreus* was performed according to EUCAST guidelines [36] (www.eucast.org, accessed on 12 January 2021). Amphotericin B (Sigma-Aldrich, Vienna, Austria A2411) (AMB), voriconazole (Sigma-Aldrich, Vienna, Austria PZ0005) (VRC), posaconazole (Sigma-Aldrich, Vienna, Austria SML2287) (POS), and caspofungin (Sigma-Aldrich, Vienna, Austria SML0425) (CAS) were evaluated.

### 2.6. Microsatellite Typing of A. terreus

*A. terreus* isolates were subjected to microsatellite typing using a panel of nine short tandem repeats (STR) to evaluate the genetic relatedness [37]. Isolates were considered to be (i) identical when all nine markers yielded corresponding repeat numbers, (ii) genetically closely related, when their genotypes differed in only one marker and (iii) unrelated if their genotypes differed in more than one marker. Bionumerics version 7.6.1 (Applied Maths, Sint-Martens-Latem, Belgium) was used to calculate the minimum spanning tree.

### 2.7. Statistical Analysis

MIC/MEC_50_, MIC_90_, MIC/MEC range and geometric mean (GM) values were calculated by Microsoft Office Excel 2016. The chi-square test was used for the assessment between isolated strains, the location of the isolates, and the collection period. *p*-values ≤ 0.05 were considered statistically significant.

## 3. Results

### 3.1. A. Terreus Collected from Environmental Samples

3485 samples from different environmental sources were collected in Tyrol, West Austria: 1714 during summer (May 2019 and October 2019) and 1771 in winter (November 2019 and April 2020). Urban and rural areas were sampled including soil (n = 1548, 44.4%), living and dead plant material (n = 580, 16.6%), air (n = 502, 14.4%), decaying material (n = 600, 17.2%), water (n = 164, 4.7%), and others (e.g., dust and excreta) (n = 91, 2.6%).

*A. fumigatus* was most frequently recovered (n = 2398/3485, 68.8%), followed by *A. flavus* (n = 889/3485, 25.5%), *A. niger* (n = 887/3485, 25.4%), and *A. terreus* (n = 189/3485, 5.4%), see Table 1. *A. terreus* was most prevalent in soil (n = 127, 67.2%), see Table 2. Detection of *A. terreus* was significantly higher (*p*-value = 0.0231) in winter (n = 121/1771, 6.8%) than in summer (n = 68/1714, 3.9%), see Table 1. Out of 189 *A. terreus* positive environmental samples, 238 *A. terreus* strains were obtained. Molecular based identification revealed the majority to be *A. terreus* and other species accounted for 5.0% (n = 12/242), being *A. citrinoterreus* (n = 3), *A. floccosus* (n = 1) and *A. hortae* (n = 1) and other species (n = 7) belonging to other sections or genera.

### 3.2. Environmental Sampling Conditions

The thermotolerant *A. terreus* species has an optimal growth temperature at 30–37 °C [38], and Tyrol has a relatively continental moderate inner-alpine mountain climate. The majority of samples were positive for *A. terreus* under the following conditions: ambient temperature below 20 °C, air humidity between 71–90%, wind speed <5 km/h, and an altitude between 500–1000m. Overall, 51.3% of *A. terreus* isolates (n = 97/189) were sampled at low (0.5–10 °C) or freezing temperatures, isolates were not collected above 30 °C. The minimum pH of 695 soil samples was 4.5, the maximum 11.0, and the actual mean was 6.9 (*p*-value < 0.0001); 87.5% (n = 63) of the soil samples showed pH-values between 6.5 and 7.5, indicating that neutral conditions favor the presence and growth of *A. terreus* in soil.

In summary, none of these climatic and environmental conditions gave a significant hint of the ecological preferences of *A. terreus* but rather indicate the ability for a wide adaptive capacity of this fungus in the environment.

### 3.3. Environmental Distribution of A. terreus

**Figure 1:**
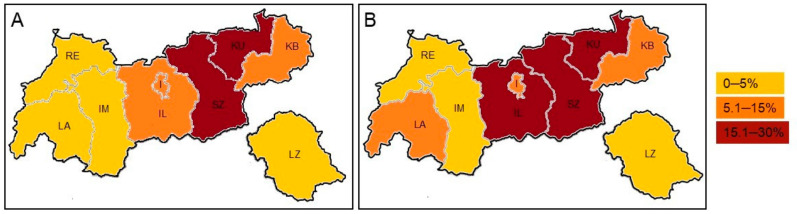
*A. terreus* prevalence in Tyrol related to the environment (**A**) and clinical cases per residence (**B**). (**A**) The highest environmental *A. terreus* frequency was detected in the districts of Schwaz (SZ, 29.6%), Kufstein (KU, 21.2%), and Innsbruck-Land (IL, 14.8%), considering the annual distribution. (**B**) Similar to the environmental distribution, most of the clinical isolates were from patients living in the districts of Kufstein (KU, 29.4%), Schwaz (SZ, 23.5%), and Innsbruck-Land (IL, 23.5%).

The federal-state Tyrol is divided into nine districts with an up-, and lowland area. In the “Tyrolean lowland,” *A. terreus* was present in 12.5% (n = 117/937) of samples, with the Zillertal valley demonstrating a frequency of 33.1% (n = 51/154). In contrast, only 3.2% (n = 22/684) were positive in the “Tyrolean upland”. In other words, from a total of 189 samples with *A. terreus*, 61.9% (n = 117) were recovered from the “Tyrolean lowland” and only 11.6% (n = 22) from the “Tyrolean upland.” Detailed information is given in Table 3 and Figure 1.

### 3.4. A. terreus Isolates from Clinical Samples

*A. terreus* isolates (n = 51) were collected from 34 patients at the Medical University Innsbruck, specimens include broncho-alveolar lavages (n = 18, 35.2%), sputum (n = 21, 41.2%), ear (n = 11, 21.6%) and wound swabs (n = 1, 1.9%). The majority of patients with *A. terreus* infection/colonization reside in lowland districts as shown in Figure 1. One *A. floccosus* (n = 1) was detected.

### 3.5. Microsatellite Typing

**Figure 2:**
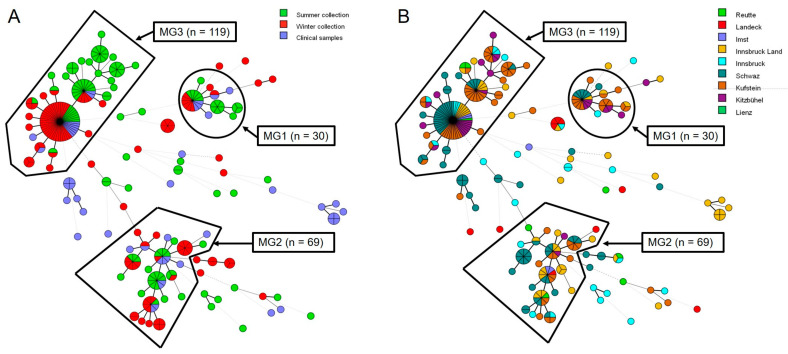
Minimum Spanning Tree (MST) of *A. terreus* environmental and clinical isolates based on microsatellite data from Tyrol, Austria. (**A**) Distribution of environmental and clinical isolates. (**B**) Distribution of all isolates in the districts of Tyrol. Black boundaries represent the three major genotypes (MG1-3), circles different genotypes. The size of each circle represents the number of isolates belonging to the same genotype. In the MST, thick and medium-thick branches indicate 1 or 2 microsatellite marker differences, respectively. The thick dashed line indicates three marker differences between two genotypes; four or more microsatellite markers differences between genotypes are indicated by medium thick and thin dashed lines, respectively.

*A. terreus* isolates (n = 289) collected from the Tyrolean environment (n = 238) and patients (n = 51) were subject to STR analysis. A total of 109 genotypes within *A. terreus* strains were identified. Results for each of the nine markers and identified genotypes are provided in Supplementary Material, Table S1. From the *A. terreus* strains investigated, 75.8% (n = 219) were assigned to three major genotype (MG1-3) clusters. Overall, 82.9% (n = 190/229) of the environmental isolates and 56.9% (n = 29/51) of the clinical isolates could be assigned to one of the three MGs (Figure 2). Clinical isolates not belonging to one of the MGs were not found in the Tyrol environment; hence, no overlap was found with the environmental samples.

### 3.6. Antifungal Susceptibility Testing

**Figure 3:**
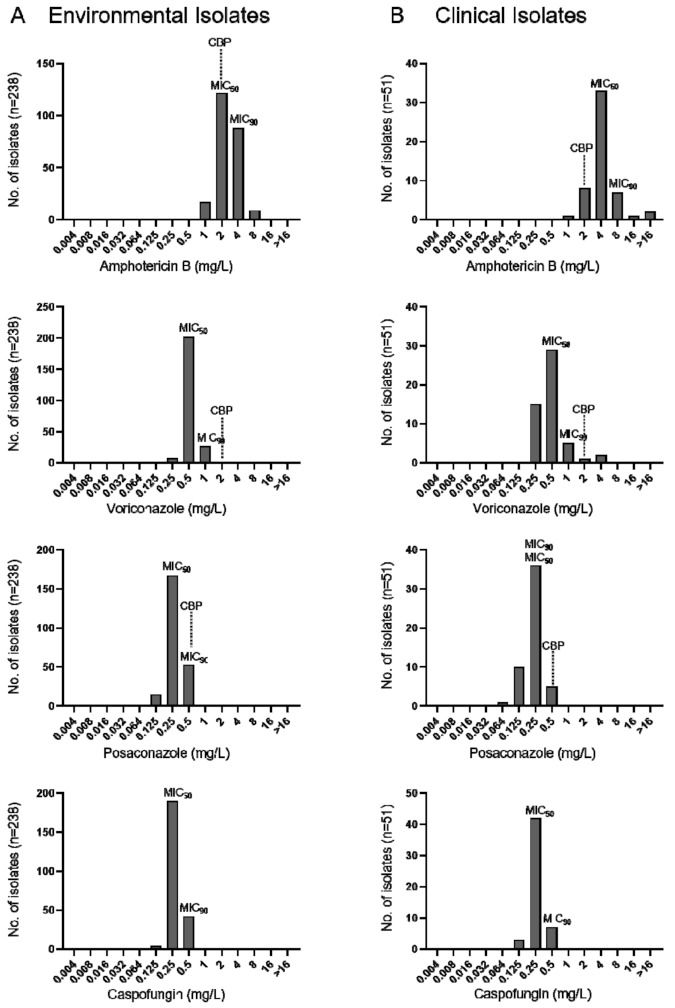
MIC distributions of amphotericin B, voriconazole, posaconazole and caspofungin against *A. terreus* obtained from environmental (**A**) and clinical (**B**) samples. Data for caspofungin are given in minimal effective concentrations (MEC) values. MIC, minimum inhibitory concentration; MIC_50_ and MIC_90_, MIC for 50 and 90% of tested population; CBP EUCAST clinical breakpoint.

The minimal inhibitory concentrations (MICs) of AMB, VRC, POS, and CAS of environmental and clinical *A. terreus* isolates are given in Figure 3. All *A. terreus* including few cryptic species showed high amphotericin B MICs ranging from 1–8 mg/mL for environmental and 2 - >16 mg/mL for clinical isolates. VRC MICs of environmental and clinical isolates ranged from 0.125–1 mg/mL and 0.250–4 mg/mL, respectively (Figure 3). According to EUCAST clinical breakpoints 5.9% (n = 3/51) of clinical *A. terreus* were classified as VRC resistant, 22.6% (n = 54/238) and 9.9% (n = 5/51) of environmental and clinical *A. terreus* were categorized as POS resistant. Cross-resistance was not noticed. Discreet were minimal effective concentrations (MEC) for CAS (0.125–1 mg/mL) in the absence of breakpoints defined. MICs are provided in Supplementary Material, Table S2.

## 4. Discussion

In the present study, *A. terreus* was identified in 5.4% (n = 189/3485) of environmental samples, indicating an increased environmental abundance in Tyrol when compared to other regions. For instance, a collection of environmental samples in Cologne and Madrid showed *A. terreus* to be positive in only 0.2% and 0.5%, respectively [38,39]. In 2008, 0.97% of air samples obtained from inside and outside the University Hospital of Innsbruck, Austria revealed *A. terreus*; In this study, 502 air samples collected throughout Tyrol demonstrated a prevalence of 4.0% of *A. terreus* (n = 20 isolates).

Our findings suggest that soil represents the primary environmental niche and a stable reservoir for this species, with a relatively high amount of *A. terreus* detected in agricultural cornfields. The reason for this finding remains unclear, but pest management applied in farming might foster the presence of *A. terreus* in this particular habitat. These data suggest that *A. terreus* does not favor complex compost and forest habitats that typically provide plant biomass as growth substrates. Besides, moisture, temperature, and biotic factors may impact fungal persistence in soil [40]. However, most common were *A. fumigatus* (68.8%), followed by *A. flavus* (25.5%), and *A. niger* (25.4%), which is largely in accordance with other studies [38,39]. It is noteworthy that *A. flavus* and *A. niger* rank 2nd and 3rd position within *Aspergillus* species identified in the environment but rarely cause infections or colonization. Hence, other factors may trigger *A. terreus* to become the 2nd most common causative agent of aspergillosis in Tyrol.

We found a significantly increased environmental *A. terreus* frequency in cold-season compared to the warm-season (*p*-value > 0.0001). Previous environmental studies point to the importance of meteorological conditions in driving airborne spore density [41,42]. Overall, 1714 and 1771 samples were collected during wet and warm, as well as dry and cold conditions; 80.3% of *A. terreus* positive samples were collected at temperatures between −10 °C to 10 °C, relative humidity between 51% to 90%, and a low wind speed. In Madrid (Spain), Guinea et al. described the highest load of *Aspergillus* spp., including *A. terreus* to be in autumn [39]. Wind speed plays an essential role in fungal spore release and dispersal. Overall, 77.8% of identified *A. terreus* isolates were collected at days with a wind speed below 10 km/h. Similar data were found by other groups for various fungal species [39,43,44]. Airborne spore abundance increases along with temperature, relative humidity, and precipitation [45,46,47]. However, these studies did not include other than air samples, which might be one reason for the deviant results found in this study. Bedsides meteorological factors, pH is an important criterion for understanding the ecology of *Aspergillus* species [48]. In this study, the majority of *A. terreus* strains were isolated from neutral to slightly acidic soil samples (pH mean 6.9). Laboratory based studies observed a maximum biomass production and sporulation of *A. terreus* at pH 5 [49]. Based on our findings, we hypothesize that a combination of various environmental conditions such as meteorological and climate factors, soil pH, the vegetation, and topographical position drive the quantity of *A. terreus* in the Tyrolean environment.

This study identified a significant high *A. terreus* prevalence in the “Tyrolean lowland” (61.9%, n = 117/189) compared to the “Tyrolean upland” (11.6%, n = 22/189). The reason for this phenomenon remains unclear. Meteorological variables, seasonal input, vegetation, topographical position, altitude, population density, agricultural holding, traffic situation, and economic establishments are comparable throughout Tyrol and therefore do not provide a clear insight. At this point, we cannot rule out if locally restricted abiotic and biotic environmental factors influence this epidemiological situation. So far, this environmental distribution pattern in Tyrol is in line with patients infected or colonized with *A. terreus*. Geographical locations with an increased environmental *A. terreus* prevalence are likely to be associated with an increased incidence of *A. terreus* infections. The fact that *A. terreus* is highly abundant in the “Tyrolean lowland” explains the frequent recovery from clinical samples in this area.

The present study addressed the antifungal susceptibility patterns of AMB, VRC, POS, and CAS against environmental and clinical *A. terreus* isolates. Clinical failure in the treatment of *A. terreus* diseases with AMB is well known [27,50,51,52]. Due to its natural resistance to polyenes, the European Committee on Antimicrobial Susceptibility Testing (EUCAST) and The Clinical Laboratory Standard Institute (CLSI) have categorized *A. terreus* as AMB resistant [53,54]. As expected, all tested *A. terreus* isolates showed high MICs; our data are in-line with other studies [4,51,55,56,57,58]. VRC resistance (MIC ≥ 2 mg/L) was observed in 3.9% (n = 2/51) of clinical isolates. In total, 22.6% of environmental (n = 54/238) and 9.8% of clinical (n = 5/51) *A. terreus* isolates were POS resistant considering a MIC > 0.250 mg/L according to EUCAST. Similar MIC values for POS in *A. terreus* were reported in other studies [23,24].

STR typing was applied for identification and typing a large number of *A. terreus* isolates collected from environmental and clinical sources in the restricted area of Tyrol, Austria. In the past, the majority of genotype studies on *A. terreus* revealed a high strain diversity among environmental and clinical isolates, indicating that patients are infected with unique strains, and nosocomial acquisition of *A. terreus* species to be unlikely [10,29,33,38]. A high degree of genetic diversity of *A. terreus* from environmental and clinical strains obtained from India, North America, and Europe was described using STR and amplified fragment length polymorphism analysis [59]. In contrast, Balajee et al. reported the existence of a single, globally distributed *A. terreus* population, and a study performed by Neal et al. using inter-simple sequence repeat PCR revealed an association between geography and genotype [60,61]. The latter is supported by a STR-genotyping study that describes three major endemic genotypes (MG1-3) collected from clinical sources in Tyrol, Austria [34]. The same three MG1-3 were found in over 75% of *A. terreus* isolates in our environmental study. The fact that all strains within the MG1-3 were of the same mating type indicates a mating barrier and is in agreement with the persistence of these genotypes in Tyrol/Austria for at least 19 years. Environmental (n = 14) and clinical (n = 12) *A. terreus* isolates, belonging to MG1-3, were randomly selected to study the mating type distributions. All strains investigated carry the *mat1-2* allele, indicating that MG1-3 were of the same mating type (data not shown). The percentage of environmental and clinical genotypes within the three clusters did not change considerably during the study period.

## 5. Conclusions

A large number of *A. terreus* isolates was found in various environmental samples in Tyrol, Austria. The environmental distribution varied within this region with a significantly higher frequency found in the “Tyrolean upland” which perfectly correlates with clinical cases and, hence, increases the risk of developing *A. terreus* infections in specific geographic locations. A favored ecological niche that explains the enhanced prevalence of *A. terreus* in certain regions might depend on a combination of meteorological factors, pH values, vegetation, and topographical positions. Most of *A. terreus* isolates investigated belong to three persisting major genotypes. This study yielded a better understanding of the molecular epidemiology of *A. terreus* in Tyrol/Austria, an area with a high *A. terreus* prevalence.

## Figures and Tables

**Table 1:** *Aspergillus* species recovered from environmental samples between May 2019 and June 2020.

*Aspergillus* Spp.	No. (%) of Positive Samples		
	All (n = 3485)	Summer (n = 1714)	Winter (n = 1771)
*Aspergillus fumigatus*	2398 (68.8)	1138 (66.4)	1260 (71.1)
*Aspergillus flavus*	889 (25.5)	531 (30.1)	358 (20.2)
*Aspergillus niger*	887 (25.4)	453 (26.4)	434 (24.5)
*Aspergillus terreus*	189 (5.4)	68 (3.9)	121 (6.8)

**Table 2:** *A. terreus* positive environmental samples, collected between May 2019 and June 2020.

Sample Material	No. (%) of Identified *A. Terreus* Species Complex		
	All	Summer	Winter
Soil	127 (67.2)	55 (80.9)	72 (59.5)
Plant material	22 (11.6)	6 (8.8)	16 (13.2)
Air	20 (10.6)	4 (5.9)	16 (13.2)
Decaying material	17 (9)	3 (4.4)	14 (11.6)
Water	2 (1.1)	0 (0)	2 (1.7)
Other	1 (0.5)	0 (0)	1 (0.8)
Total	189/3485 (5.4)	68/1714 (3.9)	121/1771 (6.8)

**Table 3:** Districts of Tyrol with identified *A. terreus* from various environmental samples.

District	No. (%) of Identified *A. terreus* Isolates		
	All (n = 189)	Summer (n = 68)	Winter (n = 121)
RE (Reutte) ^1^	8 (4.2)	4 (5.9)	4 (3.3)
LA (Landeck) ^1^	7 (3.7)	3 (4.4)	4 (3.3)
IM (Imst) ^1^	7 (3.7)	2 (2.9)	5 (4.1)
IL (Innsbruck Land)	28 (14.8)	12 (17.6)	16 (13.2)
I (Innsbruck)	21 (11.1)	6 (8.8)	15 (12.4)
SZ (Schwaz) ^2^	56 (29.6)	15 (22.1)	41 (33.9)
KU (Kufstein) ^2^	40 (21.2)	16 (23.5)	24 (19.8)
KB (Kitzbühel) ^2^	21 (11.1)	9 (13.2)	12 (9.9)
LZ (Lienz)	1 (0.5)	1 (1.5)	0 (0)

^1^ RE, LA, IM belong to the “Tyrolean upland”. ^2^ SZ, KU, KB belong to the “Tyrolean lowland”.

## Supplementary Materials

The following are available online at https://www.mdpi.com/2076-2607/9/3/539/s1, Table S1: STR analysis of environmental and clinical *A. terreus* isolates from Tyrol/Austria. Table S2: MIC-data per strain and antifungals.
