# Supplementary material for: The Environmental Spread of Aspergillus terreus in Tyrol, Austria
Source: Microorganisms. 2021 Mar 5;9(3):539. doi: 10.3390/microorganisms9030539 (PMC7998223; doi:10.3390/microorganisms9030539)
Supplement: Supplementary file 1 [file microorganisms-09-00539-s001.pdf]

Supplementary Table S1. STR analysis of environmental and clinical *A. terreus* isolates from Tyrol/Austria. Results for each of the nine markers (2A, 2B, 2C, 3A, 3B, 3C, 4A, 4B, 4C) are given together with the resulting genotype. Reference strains used are included. Values that represent -2 indicate the absence of the specific product. The absence of products was tested in multiplex and confirmed by singleplex PCR. Cryptic species belonging to the section *Terrei* are highlighted in grey. Source: environmental (E) and clinical (C).

Strain ID	Source	Identity	2A	2B	2C	3A	3B	3C	4A	4B	4C
refrerence strain		*Aspergillus terreus*	9	10	27	9	13	32	8	20	5
refrerence strain		*Aspergillus terreus*	27	8	20	8	9	29	5	8	7
refrerence strain		*Aspergillus terreus*	11	10	17	9	8	18	8	7	5
refrerence strain		*Aspergillus terreus*	11	8	19	5	10	17	10	9	5
refrerence strain		*Aspergillus terreus*	10	22	26	5	7	29	9	7	16
refrerence strain		*Aspergillus terreus*	10	12	18	9	13	21	8	9	8
refrerence strain		*Aspergillus terreus*	9	8	21	4	7	29	5	8	7
refrerence strain		*Aspergillus terreus*	10	10	21	8	8	7	10	16	8
refrerence strain		*Aspergillus hortae*	6	1	13	-2	4	-2	5	3	6
refrerence strain		*Aspergillus hortae*	6	-2	25	-2	4	-2	5	3	5
refrerence strain		*Aspergillus neoafricanus*	7	1	13	5	20	22	6	9	5
refrerence strain		*Aspergillus hortae*	6	7	13	-2	8	23	5	3	6
refrerence strain		*Aspergillus terreus*	10	10	4	10	12	18	8	8	11
refrerence strain		*Aspergillus terreus*	8	10	18	5	9	22	8	6	5
refrerence strain		*Aspergillus terreus*	19	18	24	15	21	18	9	9	8
refrerence strain		*Aspergillus terreus*	27	8	20	8	9	30	5	8	7
refrerence strain		*Aspergillus terreus*	28	8	20	8	9	32	5	8	7
refrerence strain		*Aspergillus hortae*	6	-2	1	-2	4	-2	5	3	5
refrerence strain		*Aspergillus terreus*	10	22	26	5	7	29	9	7	16
refrerence strain		*Aspergillus citrinoterreus*	6	14	8	4	7	17	5	3	4
refrerence strain		*Aspergillus citrinoterreus*	6	7	8	5	7	21	5	-2	3
refrerence strain		*Aspergillus citrinoterreus*	6	7	8	5	7	20	5	-2	4
refrerence strain		*Aspergillus terreus*	9	8	20	4	7	29	5	8	7
refrerence strain		*Aspergillus citrinoterreus*	6	7	8	5	7	17	5	3	3
refrerence strain		*Aspergillus terreus*	11	22	20	11	8	20	6	9	5
refrerence strain		*Aspergillus citrinoterreus*	6	7	8	5	7	-2	5	3	4
refrerence strain		*Aspergillus neoafricanus*	6	11	9	4	9	-2	5	6	6
refrerence strain		*Aspergillus hortae*	6	6	12	19	5	-2	5	3	5
refrerence strain		*Aspergillus terreus*	13	11	23	6	11	12	9	5	5
# 1, 11.06.2019-I-S-51, B.1/2	E	*Aspergillus terreus*	20	27	20	4	7	-2	5	5	16
# 2, 17.06.2019-I-S-33, 33.4 (1)	E	*Aspergillus terreus*	21	26	20	4	7	-2	5	5	14
# 3, 17.06.2019-I-S-33, 33.4 (2)	E	*Aspergillus terreus*	21	26	20	4	7	-2	5	5	14
# 4, 02.07.2019-KB-S-23, 23.2	E	*Aspergillus terreus*	20	26	19	4	7	-2	5	5	14
# 5, 08.07.2019-I-S-1, A 50 °C	E	*Aspergillus terreus*	20	13	12	6	6	6	5	3	5

# 6, 08.07.2019-I-S-1, B.2 50 °C	E	*Aspergillus terreus*	19	31	17	8	6	30	9	17	7
# 7, 09.07.2019-I-P-10, 10.2	E	*Aspergillus terreus*	24	7	15	8	6	36	9	14	10
# 8, 11.07.2019-IL-S-41, B AmB	E	*Aspergillus terreus*	28	8	20	8	8	30	5	8	7
# 9, 11.07.2019-IL-S-41, B.2	E	*Aspergillus terreus*	20	26	20	13	5	-2	5	5	14
# 10, 17.07.2019-I-P-21, 50 °C	E	*Aspergillus terreus*	11	8	10	4	9	15	10	10	7
# 11, 22.07.2019-KU-S-29, B AmB	E	*Aspergillus terreus*	27	8	20	8	8	30	5	8	7
# 12, 22.07.2019-KU-S-30, B.1	E	*Aspergillus terreus*	26	8	20	8	8	30	5	8	7
# 13, 22.07.2019-KU-S-32, A AmB	E	*Aspergillus terreus*	21	26	20	4	7	-2	5	5	14
# 14, 22.07.2019-KU-S-32, A.1	E	*Aspergillus terreus*	21	26	20	4	7	-2	5	5	14
# 15, 22.07.2019-KU-S-32, A.2 (1)	E	*Aspergillus terreus*	21	26	20	4	7	-2	5	5	14
# 16, 22.07.2019-KU-S-32, A.2 (2)	E	*Aspergillus terreus*	21	26	20	4	7	-2	5	5	14
# 17, 22.07.2019-KU-S-32, A.2 (3)	E	*Aspergillus terreus*	20	26	20	4	7	15	5	5	14
# 18, 22.07.2019-KU-S-32, A.2 (4)	E	*Aspergillus terreus*	21	26	20	4	7	-2	5	5	14
# 19, 24.07.2019-KB-S-11, A.1	E	*Aspergillus terreus*	21	26	20	4	7	-2	5	5	14
# 20, 24.07.2019-KB-S-22, B.2	E	*Aspergillus terreus*	13	8	18	4	13	15	9	9	7
# 21, 24.07.2019-KB-S-57, B AmB	E	*Aspergillus terreus*	13	35	18	4	13	15	9	9	7
# 22, 24.07.2019-KB-S-64, B.1	E	*Aspergillus terreus*	21	26	20	4	7	-2	5	5	16
# 23, 24.07.2019-KB-P-72, 1	E	*Aspergillus terreus*	27	8	20	8	8	30	5	8	7
# 24, 24.07.2019-KB-P-72, 2	E	*Aspergillus terreus*	29	8	20	8	8	30	5	8	7
# 25, 24.07.2019-KB-S-85, A.2	E	*Aspergillus terreus*	13	35	18	4	13	15	9	9	7
# 26, 24.07.2019-KB-S-86, A AmB	E	*Aspergillus terreus*	21	26	20	4	7	-2	5	5	14
# 27, 29.07.2019-I-S-2, A.2 (1)	E	*Aspergillus terreus*	10	15	10	5	6	12	8	4	5
# 28, 29.07.19-I-S-2-A-2, 2	E	*Aspergillus terreus*	20	31	17	8	6	30	9	17	7
# 29, 29.07.2019-I-S-2, A AmB	E	*Aspergillus terreus*	10	15	10	5	6	12	8	4	5
# 30, 29.07.2019-I-S-2, B.1 (4)	E	*Aspergillus terreus*	20	30	17	8	6	30	9	17	7
# 32, 30.07.2019-KU-S-11, A.1 (2)	E	*Aspergillus terreus*	13	35	18	4	13	15	9	9	7
# 33, 30.08.19-KU-S-11-A.1, 3	E	*Aspergillus terreus*	13	35	19	4	13	15	9	9	7
# 34, 30.07.2019-KU-S-11, A AmB (1)	E	*Aspergillus terreus*	13	35	18	4	13	15	9	9	7
# 36, 30.07.2019-KU-S-11, A.2 (1)	E	*Aspergillus terreus*	13	35	18	4	13	15	9	9	7
# 37, 30.07.2019-KU-S-11, B.1 (2)	E	*Aspergillus terreus*	13	35	18	4	13	15	9	9	7
# 38, 30.07.2019-KU-S-11, B AmB (1)	E	*Aspergillus terreus*	13	34	18	4	13	15	9	9	7
# 39, 30.07.2019-KU-S-11, B.2 (1)	E	*Aspergillus terreus*	13	34	18	4	13	15	9	9	7
# 40, 30.07.2019-KU-S-18, B.1 (2)	E	*Aspergillus terreus*	20	25	20	4	7	-2	5	5	14
# 41, 30.07.2019-KU-S-18, B AmB (1)	E	*Aspergillus terreus*	20	25	20	4	7	-2	5	5	14
# 42, 30.07.2019-KU-S-18, A.2	E	*Aspergillus terreus*	20	25	20	4	7	-2	5	5	14
# 43, 30.07.2019-KU-S-18, A AmB	E	*Aspergillus terreus*	20	25	20	4	7	-2	5	5	14
# 44, 30.07.2019-KU-S-21, A AmB (1)	E	*Aspergillus terreus*	20	25	20	4	7	-2	5	5	14
# 45, 30.07.2019-KU-S-21, A.2	E	*Aspergillus terreus*	20	25	20	4	7	-2	5	5	14
# 46, 30.07.2019-KU-S-21, A.1 (1)	E	*Aspergillus terreus*	20	25	20	50	7	-2	5	5	14
# 47, 30.07.2019-SZ-S-45, A AmB (1)	E	*Aspergillus terreus*	20	25	20	4	7	-2	5	5	14
# 48, 30.07.2019-SZ-S-45, B AmB (1)	E	*Aspergillus terreus*	27	8	20	8	9	32	5	8	7
# 49, 30.07.2019-KU-DM-101, 2	E	*Aspergillus terreus*	20	25	20	4	6	-2	5	5	14
# 50, 01.08.2019-IL-S-7, A.2	E	*Aspergillus terreus*	13	34	18	4	13	15	9	9	7

# 51, 01.08.2019-IL-S-7, A	E	*Aspergillus terreus*	18	22	21	4	8	-2	9	5	7
# 52, 07.08.2019-RE-S-21, A.2	E	*Aspergillus terreus*	20	25	20	4	6	-2	5	5	14
# 52,): 01.08.19-IL-S-7-A	E	*Aspergillus terreus*	15	8	18	8	7	26	8	9	5
# 53, 07.08.2019-RE-S-35, B.1	E	*Aspergillus terreus*	10	5	10	7	7	33	8	3	5
# 54, 07.08.2019-RE-S-52, 1	E	*Aspergillus terreus*	11	8	20	4	8	-2	10	6	7
# 55, 07.08.2019-RE-A-81	E	*Aspergillus terreus*	20	25	20	4	6	-2	5	5	14
# 56, 12.08.2019-IL-S-27, B AmB	E	*Aspergillus terreus*	20	25	20	4	6	-2	5	5	14
# 57, 20.08.2019-IL-S-11, B.2 (1)	E	*Aspergillus terreus*	28	8	20	8	9	29	5	8	7
# 58, 22.08.2019-LA-S-55, B.2	E	*Aspergillus terreus*	27	8	20	8	8	32	5	8	7
# 59, 22.08.2019-LA-S-62, A.2	E	*Aspergillus terreus*	1	15	14	5	5	9	6	8	6
# 60, 27.08.2019-IL-S-5, B.1	E	*Aspergillus terreus*	13	8	13	5	14	18	8	7	5
# 61, 27.08.2019-IL-S-22, A.1	E	*Aspergillus terreus*	20	26	21	4	6	-2	5	5	14
# 62, 27.08.2019-IL-A-25	E	*Aspergillus terreus*	28	8	21	8	8	30	5	8	7
# 63, 27.08.2019-IL-S-28, A AmB	E	*Aspergillus terreus*	20	26	20	4	5	10	5	5	14
# 64, 27.08.2019-IL-S-54, A.1	E	*Aspergillus terreus*	27	8	21	8	8	32	5	8	7
# 65, 27.08.2019-IL-S-57, A AmB	E	*Aspergillus terreus*	20	26	21	4	6	-2	5	5	14
# 66, 27.08.2019-LZ-S-67, A.1	E	*Aspergillus terreus*	11	8	10	4	8	15	10	10	7
# 67, 27.08.2019-KB-S/P-86, 2	E	*Aspergillus terreus*	20	26	20	4	7	-2	5	5	14
# 68, 03.09.2019-LA-S-14, B.2	E	*Aspergillus terreus*	11	10	18	5	10	13	8	10	7
# 69, 03.09.2019-SZ-S-22, A + AmB	E	*Aspergillus terreus*	20	26	21	4	6	-2	5	5	14
# 70, 03.09.2019-SZ-S-22, A.1	E	*Aspergillus terreus*	20	26	21	4	6	-2	5	5	14
# 71, 03.09.2019-SZ-S-22, A.2	E	*Aspergillus terreus*	27	8	21	8	8	31	5	8	7
# 72, 03.09.2019-SZ-S-26, A + AmB	E	*Aspergillus terreus*	20	26	21	4	6	-2	5	5	14
# 73, 03.09.2019-SZ-S-29, A.1	E	*Aspergillus terreus*	13	35	19	4	13	15	9	9	7
# 74, 03.09.2019-SZ-S-34, A.2	E	*Aspergillus terreus*	20	26	20	4	6	-2	5	5	14
# 75, 03.09.2019-SZ-S-37, B.2	E	*Aspergillus terreus*	28	8	20	8	8	32	5	8	7
# 76, 03.09.2019-SZ-S-37, A + AmB	E	*Aspergillus terreus*	13	35	19	4	13	15	9	9	7
# 77, 03.09.2019-SZ-S-43, A + AmB	E	*Aspergillus terreus*	22	26	21	8	6	32	5	7	7
# 78, 03.09.2019-SZ-S-43, B.2	E	*Aspergillus terreus*	22	26	21	8	6	32	5	7	7
# 79, 03.09.2019-SZ-S-44, A.2	E	*Aspergillus terreus*	13	35	19	4	13	15	9	9	7
# 80, 03.09.2019-SZ-S-47, A + AmB	E	*Aspergillus terreus*	20	26	20	4	6	15	5	5	14
# 81, 03.09.2019-SZ-S-47, A.2	E	*Aspergillus terreus*	20	26	21	4	6	-2	5	5	14
# 82, 03.09.2019-SZ-S-47, A.1	E	*Aspergillus terreus*	20	26	21	4	6	-2	5	5	14
# 83, 03.09.2019-SZ-S-47, B.1 (1)	E	*Aspergillus terreus*	21	26	21	4	7	-2	5	5	14
# 84, 03.09.2019-SZ-S-47, B.1 (2)	E	*Aspergillus terreus*	27	8	21	8	8	31	5	8	7
# 85, 03.09.2019-SZ-P-59, +AmB	E	*Aspergillus terreus*	20	26	21	4	7	-2	5	5	14
# 86, 03.09.2019-SZ-P-59, 2	E	*Aspergillus terreus*	20	26	20	4	6	-2	5	5	14
# 87, 03.09.2019-SZ-P-65, +AmB	E	*Aspergillus terreus*	21	26	21	4	6	-2	5	5	14
# 88, 03.09.2019-SZ-S-67, A.1	E	*Aspergillus terreus*	20	26	21	4	6	-2	5	5	14
# 89, 03.09.2019-SZ-S-67, B.1	E	*Aspergillus terreus*	27	8	21	8	8	31	5	8	7
# 90, 03.09.2019-SZ-S-69, B.1	E	*Aspergillus terreus*	25	26	22	8	6	32	5	7	7
# 91, 03.09.2019-SZ-S-71, A.2	E	*Aspergillus terreus*	28	8	21	8	8	31	5	8	7
# 92, 03.09.2019-SZ-S-71, B + AmB	E	*Aspergillus terreus*	27	8	20	8	8	32	5	8	7

# 93, 03.09.2019-SZ-S-73, A + AmB	E	*Aspergillus terreus*	27	8	20	8	8	32	5	8	7
# 94, 17.06.2019-IL-A-AstridM-out, 1	E	*Aspergillus terreus*	20	26	20	4	6	-2	5	5	14
# 95, 17.06.2019-IL-A-AstridM-out, 2	E	*Aspergillus terreus*	20	26	20	4	6	-2	5	5	14
# 96, 17.06.2019-IL-A-MichiM-out, 1	E	*Aspergillus terreus*	27	8	20	8	8	32	5	8	7
# 97, 30.07.2019-SZ-S-45, A.2	E	*Aspergillus terreus*	20	26	20	4	6	-2	5	5	14
# 98, 10.09.2019-IM-S-14, A 50 °C (1)	E	*Aspergillus terreus*	27	8	20	8	8	32	5	8	7
# 99, 10.09.2019-IM-S-40, B.2 (2)	E	*Aspergillus terreus*	27	8	20	8	8	32	5	8	7
# 100, 19.09.2019-IL-S-13, B 50 °C (1)	E	*Aspergillus terreus*	28	8	20	8	8	30	5	8	7
# 101, 19.09.2019-IL-S-13, B 50 °C (2)	E	*Aspergillus terreus*	27	8	20	8	8	32	5	8	7
# 102, 19.09.2019-IL-S-13, B AmB (1)	E	*Aspergillus terreus*	27	8	20	8	8	30	5	8	7
# 103, 19.09.2019-IL-S-13, A 50 °C	E	*Aspergillus terreus*	27	8	20	8	8	32	5	8	7
# 104, 19.09.2019-IL-S/DM-29, A AmB	E	*Aspergillus terreus*	20	26	20	4	6	-2	5	5	14
# 106, 25.10.2019-KU-S-12, A.2	E	*Aspergillus terreus*	13	35	19	4	13	15	9	9	7
# 107, 25.10.2019-KU-S-12, A.1 + AmB (1)	E	*Aspergillus terreus*	20	26	20	4	6	-2	5	5	16
# 108, 25.10.2019-KU-S-12, A.1 + AmB (2)	E	*Aspergillus terreus*	20	26	20	4	6	-2	5	5	16
# 109, 25.10.2019-KU-S-12, A 50°C (1)	E	*Aspergillus terreus*	27	8	20	8	8	30	5	8	7
# 110, 25.10.2019-KU-S-12, B 50°C (2)	E	*Aspergillus terreus*	20	26	20	4	6	-2	5	5	14
# 111, 25.10.2019-KU-S-17, A.2	E	*Aspergillus terreus*	20	26	20	4	6	-2	5	5	14
# 112, 25.10.2019-KU-S-21, B + AmB	E	*Aspergillus terreus*	20	26	20	4	6	-2	5	5	14
# 113, 25.10.2019-KU-S-24, A + AmB	E	*Aspergillus terreus*	20	26	22	4	6	-2	5	5	14
# 114, 25.10.2019-KU-S-30, B + AmB	E	*Aspergillus terreus*	20	26	20	4	6	-2	5	5	14
# 115, 25.10.2019-KU-P-31, 50°C	E	*Aspergillus terreus*	20	26	20	4	6	-2	5	5	14
# 116, 25.10.2019-KU-A-40	E	*Aspergillus terreus*	13	35	19	4	16	15	9	9	7
# 117, 25.10.2019-KU-A-51	E	*Aspergillus terreus*	18	22	22	-2	8	-2	9	5	7
# 118,19,01,20 KB-A-1	E	*Aspergillus terreus*	20	26	22	4	6	-2	5	5	14
# 119,19,01,20 KB-A-2	E	*Aspergillus terreus*	20	26	21	4	6	-2	5	5	16
# 120,19,01,20 KB-A-1	E	*Aspergillus terreus*	20	26	22	4	6	-2	5	5	14
# 121,19,01,20 KB-A-2	E	*Aspergillus terreus*	20	26	21	4	6	-2	5	5	16
# 121a,19,01,20 KB-A-2	E	*Aspergillus terreus*	20	26	21	4	6	-2	5	5	16
# 122 | 17.01.2020-IL-DM-12 | AmB	E	*Aspergillus terreus*	27	8	20	8	8	31	5	8	7
# 123 | 17.01.2020-IL-S-17 | A.2	E	*Aspergillus terreus*	20	26	21	4	6	-2	5	5	14
# 124 | 17.01.2020-SZ-S-41 | B.2	E	*Aspergillus terreus*	20	26	21	4	6	-2	5	5	14
# 126 | 17.01.2020-KU-S-66 | B.2	E	*Aspergillus terreus*	20	26	21	4	6	-2	5	5	14
# 127 | 17.01.2020-KU-A-68 | -1	E	*Aspergillus terreus*	27	8	21	8	8	30	5	8	7
# 128 | 17.01.2020-KU-A-74 | -1	E	*Aspergillus terreus*	20	26	21	4	6	-2	5	5	14
# 129 | 17.01.2020-KU-A-77 |	E	*Aspergillus terreus*	20	26	22	4	6	-2	5	5	14
# 130 | 17.01.2020-KU-A-83 |	E	*Aspergillus terreus*	27	8	21	8	8	32	5	8	7
# 131 | 17.01.2020-KU-A-87 |	E	*Aspergillus terreus*	11	8	10	4	8	15	10	10	7
# 132 | 17.01.2020-KU-A-92 |	E	*Aspergillus terreus*	27	8	21	8	8	-2	5	8	7
# 133 | 17.01.2020-KU-S-94 | A.1	E	*Aspergillus terreus*	27	8	21	8	8	32	5	8	7
# 134 | 17.01.2020-KU-S-106 | A AmB	E	*Aspergillus terreus*	27	8	21	8	8	30	5	8	7
# 135 | 23.01.2020-SZ-S-1 | A.1	E	*Aspergillus terreus*	27	8	21	8	8	30	5	8	7
# 136 | 23.01.2020-SZ-S-2 | A.1	E	*Aspergillus terreus*	20	26	21	4	6	-2	5	5	14

# 137 ǀ 23.01.2020-SZ-S-7 ǀ B.2	E	*Aspergillus terreus*	27	8	21	8	8	30	5	8	7
# 138 ǀ 23.01.2020-SZ-S-8 ǀ B.1	E	*Aspergillus terreus*	13	35	19	4	13	15	9	9	7
# 139 ǀ 23.01.2020-SZ-DM-9 ǀ 1 (1)	E	*Aspergillus terreus*	27	8	21	8	8	31	5	8	7
# 140 ǀ 23.01.2020-SZ-DM-12 ǀ 2	E	*Aspergillus terreus*	20	26	21	4	6	-2	5	5	14
# 141 ǀ 23.01.2020-SZ-A-13 ǀ	E	*Aspergillus terreus*	20	27	21	4	6	-2	5	5	16
# 142 ǀ 23.01.2020-SZ-S-14 ǀ B.1	E	*Aspergillus terreus*	21	26	21	4	6	-2	5	5	14
# 143 ǀ 23.01.2020-SZ-DM-16 ǀ 2	E	*Aspergillus terreus*	9	8	21	4	6	30	5	8	7
# 144 ǀ 23.01.2020-SZ-P-18 ǀ AmB	E	*Aspergillus terreus*	20	26	21	4	6	-2	5	5	14
# 145 ǀ 23.01.2020-SZ-P-19 ǀ 2	E	*Aspergillus terreus*	21	26	21	4	6	-2	5	5	17
# 146 ǀ 23.01.2020-SZ-S-23 ǀ A.2	E	*Aspergillus terreus*	27	8	21	8	8	33	5	8	7
# 147 ǀ 23.01.2020-SZ-DM-24 ǀ 1	E	*Aspergillus terreus*	20	26	21	4	7	-2	5	5	14
# 148 ǀ 23.01.2020-SZ-DM-25 ǀ 2	E	*Aspergillus terreus*	9	8	21	4	6	30	5	8	7
# 149 ǀ 23.01.2020-SZ-A-26 ǀ -1	E	*Aspergillus terreus*	20	26	21	4	6	-2	5	5	14
# 150 ǀ 23.01.2020-SZ-S-31 ǀ B.2	E	*Aspergillus terreus*	20	26	21	4	6	-2	5	5	14
# 151 ǀ 23.01.2020-SZ-A-33 ǀ -1	E	*Aspergillus terreus*	20	26	21	4	6	-2	5	5	14
# 152 ǀ 23.01.2020-SZ-S-34 ǀ B AmB	E	*Aspergillus terreus*	20	26	21	4	6	-2	5	5	17
# 153 ǀ 23.01.2020-SZ-P-36 ǀ 2	E	*Aspergillus terreus*	27	8	21	8	8	32	5	8	7
# 154 ǀ 23.01.2020-SZ-A-37 ǀ -1	E	*Aspergillus terreus*	27	8	21	8	8	30	5	8	7
# 155 ǀ 23.01.2020-SZ-S-41 ǀ B.2	E	*Aspergillus terreus*	27	8	21	8	8	31	5	8	7
# 156 ǀ 23.01.2020-SZ-P-42 ǀ AmB	E	*Aspergillus terreus*	20	26	21	4	6	-2	5	5	14
# 157 ǀ 23.01.2020-SZ-A-46 ǀ -1	E	*Aspergillus terreus*	28	8	21	8	8	32	5	8	7
# 158 ǀ 23.01.2020-SZ-S-48 ǀ A.1	E	*Aspergillus terreus*	27	8	20	8	8	30	5	8	7
# 159 ǀ 23.01.2020-SZ-S-54 ǀ A AmB (2)	E	*Aspergillus terreus*	27	8	21	8	8	31	5	8	7
# 160 ǀ 23.01.2020-SZ-S-56 ǀ B AmB	E	*Aspergillus terreus*	29	8	20	8	8	33	10	8	7
# 161 ǀ 23.01.2020-SZ-S-62 ǀ A.1	E	*Aspergillus terreus*	13	35	19	8	13	15	9	9	7
# 162 ǀ 23.01.2020-SZ-P-63 ǀ 1	E	*Aspergillus terreus*	20	26	20	4	6	-2	5	5	14
# 163 ǀ 23.01.2020-SZ-DM-64 ǀ 1 (1)	E	*Aspergillus terreus*	13	35	19	4	13	15	9	9	7
# 164 ǀ 23.01.2020-SZ-DM-65 ǀ AmB	E	*Aspergillus terreus*	27	8	21	8	8	31	5	8	7
# 165 ǀ 23.01.2020-SZ-S-66 ǀ B.2 (1)	E	*Aspergillus terreus*	20	26	21	4	6	-2	5	5	14
# 166 ǀ 23.01.2020-SZ-S-67 ǀ A.1	E	*Aspergillus terreus*	20	26	21	4	6	-2	5	5	14
# 167 ǀ 23.01.2020-SZ-P-68 ǀ 1 (1)	E	*Aspergillus terreus*	20	26	21	4	6	-2	5	5	14
# 168 ǀ 23.01.2020-SZ-A-73 ǀ -1	E	*Aspergillus terreus*	27	8	21	8	8	31	5	8	7
# 169 ǀ 23.01.2020-SZ-P-76 ǀ 1 (1)	E	*Aspergillus terreus*	9	8	20	4	6	30	5	8	7
# 170 ǀ 23.01.2020-SZ-S-87 ǀ B.2	E	*Aspergillus terreus*	20	26	21	4	6	-2	5	5	14
# 171 ǀ 23.01.2020-SZ-S-93 ǀ B.1	E	*Aspergillus terreus*	28	8	21	8	8	32	5	8	7
# 172 ǀ 30.01.2020-KU-P-12 ǀ AmB	E	*Aspergillus terreus*	13	35	19	4	13	15	9	9	7
# 173 ǀ 30.01.2020-KU-S-19 ǀ A.1	E	*Aspergillus terreus*	20	26	21	4	6	-2	5	5	14
# 174 ǀ 30.01.2020-KU-S-31 ǀ A.1	E	*Aspergillus terreus*	20	26	21	4	6	-2	5	5	14
# 175 ǀ 30.01.2020-KU-DM-34 ǀ 50°C	E	*Aspergillus terreus*	27	8	21	8	8	30	5	8	7
# 176 ǀ 30.01.2020-KU-S-41 ǀ A.1 (2)	E	*Aspergillus terreus*	27	8	21	8	8	30	5	8	7
# 177 ǀ 30.01.2020-KU-P-45 ǀ AmB	E	*Aspergillus terreus*	20	26	21	4	6	-2	5	5	14
# 178 ǀ 30.01.2020-SZ-DM/P-72 ǀ 1	E	*Aspergillus terreus*	20	26	21	4	6	-2	5	5	14
# 179 ǀ 30.01.2020-SZ-P-75 ǀ AmB (1)	E	*Aspergillus terreus*	20	27	21	4	6	-2	5	5	16

# 180 ǀ 06.02.2020-IL-A-8 ǀ	E	*Aspergillus terreus*	20	26	21	4	6	-2	5	5	14
# 181 ǀ 06.02.2020-IM-S-57 ǀ A.1	E	*Aspergillus citrinoterreus*	6	7	7	5	7	-2	5	3	4
# 182 ǀ 06.02.2020-IM-S-62 ǀ B AmB	E	*Aspergillus terreus*	20	26	21	4	6	-2	5	5	14
#183 ǀ 14.02.2020-IL-S-21 ǀ B.2	E	*Aspergillus terreus*	13	22	19	4	8	15	10	9	5
#184 ǀ 14.02.2020-IL-DM-30 ǀ 1	E	*Aspergillus citrinoterreus*	6	7	7	5	7	-2	5	3	4
#186 ǀ 20.02.2020-I-S-43 ǀ B.1	E	*Aspergillus terreus*	20	26	21	4	6	-2	5	5	14
#187 ǀ 27.02.2020-I-W-4 ǀ 2	E	*Aspergillus terreus*	20	26	22	4	6	-2	5	5	14
#188 ǀ 05.03.2020-KU-S-7 ǀ A.1	E	*Aspergillus terreus*	20	26	21	4	6	-2	5	5	5
#189 ǀ 05.03.2020-KU-S-15 ǀ A AmB	E	*Aspergillus terreus*	27	8	21	8	8	33	5	8	7
#190 ǀ 05.03.2020-KU-P-17 ǀ 1	E	*Aspergillus terreus*	27	8	21	8	8	33	5	8	7
#191 ǀ 05.03.2020-KU-DM-20 ǀ AmB	E	*Aspergillus terreus*	30	8	20	8	8	31	5	8	7
#192 ǀ 05.03.2020-KU-S-24 ǀ B.2	E	*Aspergillus terreus*	20	26	20	4	6	-2	5	5	14
#193 ǀ 05.03.2020-KU-S-27 ǀ A.2	E	*Aspergillus terreus*	20	26	20	4	6	-2	5	5	14
#194 ǀ 05.03.2020-KU-S-35 ǀ A.2	E	*Aspergillus terreus*	21	26	20	4	6	-2	5	5	14
#195 ǀ 05.03.2020-KU-P-42 ǀ 50°C	E	*Aspergillus terreus*	13	35	19	4	13	16	9	9	7
#196 ǀ 05.03.2020-KU-S-43 ǀ A AmB	E	*Aspergillus terreus*	20	26	21	4	6	-2	5	5	14
#197 ǀ 05.03.2020-KU-P-58 ǀ 1	E	*Aspergillus terreus*	20	27	21	4	6	-2	5	5	16
#198 ǀ 05.03.2020-KU-S-59 ǀ A AmB	E	*Aspergillus terreus*	20	26	21	4	6	-2	5	5	14
#199 ǀ 05.03.2020-KB-W-77 ǀ 1	E	*Aspergillus terreus*	20	26	20	4	6	-2	5	5	14
#200 ǀ 05.03.2020-I-S-101 ǀ A AmB	E	*Aspergillus terreus*	13	10	21	8	8	14	9	9	5
#201 ǀ 11.03.2020-IL-DM-7 ǀ AmB	E	*Aspergillus terreus*	28	8	20	8	8	30	5	8	7
#202 ǀ 11.03.2020-IL-S-29 ǀ B.2	E	*Aspergillus terreus*	14	9	23	11	6	92	8	13	5
#203 ǀ 15.04.2020-I-S-18 ǀ B.2	E	*Aspergillus hortae*	7	4	10	6	-2	-2	5	3	4
#204 ǀ 15.04.2020-I-S-20 ǀ A AmB	E	*Aspergillus terreus*	13	8	17	8	6	47	8	10	5
#205 ǀ 15.04.2020-I-S-74 ǀ A.2	E	*Aspergillus terreus*	22	26	22	8	6	31	5	8	7
#206 ǀ 15.04.2020-KB-A-6 ǀ	E	*Aspergillus terreus*	13	35	19	4	13	15	9	9	7
#207 ǀ 15.04.2020-KB-S-15 ǀ A.1	E	*Aspergillus terreus*	20	26	21	4	6	15	5	5	14
#208 ǀ 15.04.2020-KB-E-18 ǀ 1	E	*Aspergillus terreus*	20	26	21	4	6	-2	5	5	14
#209 ǀ 15.04.2020-KB-S-25 ǀ A AmB (1)	E	*Aspergillus terreus*	13	35	19	4	6	10	11	19	7
#210 ǀ 15.04.2020-KB-S-60 ǀ B 50°C	E	*Aspergillus terreus*	20	26	21	4	6	-2	5	5	14
#211 ǀ 15.04.2020-KB-S-65 ǀ A.1	E	*Aspergillus terreus*	13	35	19	4	13	15	9	9	7
#212 ǀ 15.04.2020-KB-S-75 ǀ A 50°C	E	*Aspergillus terreus*	20	26	21	-2	6	9	5	5	14
#213 ǀ 15.04.2020-KB-S-83 ǀ B AmB	E	*Aspergillus terreus*	20	26	21	4	6	-2	5	5	14
#214 ǀ 15.04.2020-KB-S-93 ǀ A.1	E	*Aspergillus terreus*	13	35	19	4	13	15	9	9	7
#215 ǀ 15.04.2020-KB-S-94 ǀ A.1	E	*Aspergillus terreus*	20	26	21	4	6	-2	5	5	14
#216 ǀ 17.04.2020-I-A-39 ǀ	E	*Aspergillus terreus*	20	26	21	4	6	-2	5	5	16
#217 ǀ 17.04.2020-I-S-58 ǀ B AmB	E	*Aspergillus floccosus*	18	-2	20	4	2	-2	11	3	46
#218 ǀ 17.04.2020-I-S-87 ǀ A AmB	E	*Aspergillus terreus*	9	8	21	4	6	10	5	8	7
#219 ǀ 15.04.2020-KB-A-9 ǀ	E	*Aspergillus terreus*	20	26	21	4	6	-2	5	5	14
#220 ǀ 20.04.2020-RE-S-34 ǀ B.2	E	*Aspergillus terreus*	20	26	21	4	6	-2	5	5	14
#221 ǀ 20.04.2020-RE-S-54 ǀ A AmB	E	*Aspergillus terreus*	27	8	22	8	8	31	5	8	7
#222 ǀ 20.04.2020-RE-S-94 ǀ B.1	E	*Aspergillus terreus*	27	8	21	8	8	32	5	8	7
#224 ǀ 22.04.2020-I-S-11 ǀ B.1	E	*Aspergillus terreus*	18	23	21	4	7	11	10	7	7

#225 ∣ 22.04.2020-I-S-34 ∣ B.2 (2)	E	*Aspergillus terreus*	20	26	21	4	6	-2	5	5	14
#226 ∣ 22.04.2020-I-S-49 ∣ B.1	E	*Aspergillus terreus*	22	26	21	8	6	46	5	5	7
#227 ∣ 23.04.2020-IM-P-20 ∣ 2 (1)	E	*Aspergillus citrinoterreus*	6	7	7	5	7	-2	5	3	4
#228 ∣ 23.04.2020-IM-DM-86 ∣ 2	E	*Aspergillus terreus*	10	8	18	6	6	-2	8	14	5
#229 ∣ 29.04.2020-I-S-31 ∣ B.1	E	*Aspergillus terreus*	26	8	21	8	8	32	5	8	7
#230 ∣ 29.04.2020-I-S-48 ∣ B.2	E	*Aspergillus terreus*	27	8	21	8	8	33	5	8	7
#231 ∣ 20.04.2020-RE-S-84 ∣ B.2	E	*Aspergillus terreus*	9	8	21	4	6	10	5	8	7
#232 ∣ 30.04.2020-IM-S-5 ∣ B.2	E	*Aspergillus terreus*	20	26	21	4	6	-2	5	5	14
#233 ∣ 30.04.2020-LA-P-14 ∣ 1	E	*Aspergillus terreus*	18	23	21	4	7	11	10	7	7
#234 ∣ 30.04.2020-LA-S-29 ∣ B.2	E	*Aspergillus terreus*	18	23	21	4	7	11	10	7	7
#235 ∣ 30.04.2020-LA-S-32 ∣ A.1	E	*Aspergillus terreus*	18	23	21	4	7	11	10	7	7
#236 ∣ 30.04.2020-LA-S-77 ∣ B.2	E	*Aspergillus terreus*	18	23	21	4	7	11	10	7	7
#237 ∣ 08.05.2020-IL-S-26 ∣ B AmB	E	*Aspergillus terreus*	13	35	19	4	6	11	11	19	7
#238 ∣ 08.05.2020-IL-S-40 ∣ B.1	E	*Aspergillus terreus*	20	26	21	4	6	-2	5	5	14
#239 ∣ 08.05.2020-IL-S-53 ∣ A.1 (1)	E	*Aspergillus terreus*	9	8	21	4	6	10	5	8	7
#240 ∣ 08.05.2020-IL-P-54 ∣ 1 (1)	E	*Aspergillus terreus*	27	8	21	8	8	32	5	8	7
#241 ∣ 08.05.2020-IL-P-54 ∣ AmB (3)	E	*Aspergillus terreus*	20	26	21	4	6	-2	5	5	14
#242 ∣ 08.05.2020-IL-S-80 ∣ A AmB	E	*Aspergillus terreus*	18	23	21	4	7	11	10	7	7
# 1 C.I. ∣ 6 ∣ 17047218	C	*Aspergillus terreus*	11	19	20	11	7	-2	10	9	5
# 2 C.I. ∣ 11 ∣ 17052729	C	*Aspergillus terreus*	20	26	21	4	6	-2	5	5	16
# 3 C.I. ∣ 14 ∣ 17059115	C	*Aspergillus terreus*	11	26	44	4	9	-2	5	7	13
# 4 C.I. ∣ 17 ∣ 17059743	C	*Aspergillus terreus*	20	26	21	4	6	-2	5	5	14
# 5 C.I. ∣ 31 ∣ 17078074	C	*Aspergillus terreus*	20	17	10	5	4	78	5	3	4
# 7 C.I. ∣ 74 ∣ 17128373	C	*Aspergillus terreus*	20	26	21	4	6	-2	5	5	14
# 8 C.I. ∣ 75 ∣ 17138738	C	*Aspergillus terreus*	5	12	42	4	16	-2	8	13	8
# 9 C.I. ∣ 80 ∣ 17146658	C	*Aspergillus terreus*	5	12	42	4	17	-2	8	13	8
# 10 C.I. ∣ 82 ∣ 17150971	C	*Aspergillus terreus*	11	26	44	4	9	-2	5	7	13
# 11 C.I. ∣ 83 ∣ 17151838	C	*Aspergillus terreus*	11	26	44	4	9	-2	5	7	13
# 12 C.I. ∣ 91 ∣ 17168469	C	*Aspergillus terreus*	11	26	44	4	9	15	5	7	13
# 13 C.I. ∣ 94 ∣ 17174588	C	*Aspergillus terreus*	13	34	19	4	13	15	9	9	7
# 14 C.I. ∣ 95 ∣ 17175347	C	*Aspergillus terreus*	11	26	43	4	9	-2	5	7	12
# 15 C.I. ∣ 96 ∣ 18004803	C	*Aspergillus floccosus*	20	-2	20	4	2	-2	9	3	3
# 17 C.I. ∣ 100 ∣ 18005380	C	*Aspergillus terreus*	30	8	21	8	9	30	5	8	7
# 18 C.I. ∣ 110 ∣ 18026722	C	*Aspergillus terreus*	27	8	21	8	8	32	5	8	7
# 20 C.I. ∣ 132 ∣ 18044324	C	*Aspergillus terreus*	20	26	21	4	6	-2	5	5	14
# 21 C.I. ∣ 139 ∣ 18053289	C	*Aspergillus terreus*	5	12	42	5	17	-2	8	13	8
# 22 C.I. ∣ 150 ∣ 18061520	C	*Aspergillus terreus*	20	26	21	4	6	-2	5	5	14
# 23 C.I. ∣ 186 ∣ 18091581	C	*Aspergillus terreus*	20	26	21	4	6	-2	5	5	14
# 25 C.I. ∣ 254 ∣ 18162048	C	*Aspergillus terreus*	20	26	21	4	6	-2	5	5	14
# 26 C.I. ∣ 290 ∣ 18183985	C	*Aspergillus terreus*	27	8	21	8	8	32	5	8	7
# 27 C.I. ∣ 293 ∣ 18184864	C	*Aspergillus terreus*	13	35	19	4	13	15	9	9	7
# 28 C.I. ∣ 294 ∣ 18187074	C	*Aspergillus terreus*	5	12	42	5	17	-2	8	13	8
# 30 C.I. ∣ 300 ∣ 18194956	C	*Aspergillus terreus*	13	35	19	4	6	16	9	9	7

# 31 C.I. ǀ 312 ǀ 19012080	C	*Aspergillus terreus*	10	5	27	9	6	9	8	7	7
# 33 C.I. ǀ 317 ǀ 19014800	C	*Aspergillus terreus*	25	10	20	9	9	-2	9	7	8
# 34 C.I. ǀ 319 ǀ 19016611	C	*Aspergillus terreus*	11	26	44	4	9	-2	5	7	13
# 35 C.I. ǀ 320 ǀ 19018959	C	*Aspergillus terreus*	14	12	18	11	6	23	8	8	5
# 36 C.I. ǀ 334 ǀ 19030457	C	*Aspergillus terreus*	20	26	21	4	6	-2	5	5	14
# 38 C.I. ǀ 349 ǀ 19043102	C	*Aspergillus terreus*	20	27	21	4	6	-2	5	5	14
# 39 C.I. ǀ 351 ǀ 19045986	C	*Aspergillus terreus*	28	8	20	8	8	31	5	8	7
# 40 C.I. ǀ 353 ǀ 19049535	C	*Aspergillus terreus*	13	35	19	4	13	15	9	9	7
# 43 C.I. ǀ 373 ǀ 19072054	C	*Aspergillus terreus*	5	12	42	4	21	-2	8	15	8
# 44 C.I. ǀ 375 ǀ 19075637	C	*Aspergillus terreus*	11	8	19	4	8	12	10	10	7
# 45 C.I. ǀ 379 ǀ 19092835	C	*Aspergillus terreus*	27	8	20	8	8	12	5	8	7
# 46 C.I. ǀ 380 ǀ 19097852	C	*Aspergillus terreus*	5	12	42	5	17	-2	8	13	8
# 51 C.I. ǀ 396 ǀ 19178907	C	*Aspergillus terreus*	13	35	19	4	13	16	9	9	7
# 53 C.I. ǀ 398 ǀ 20005555	C	*Aspergillus terreus*	27	8	20	8	8	30	5	8	7
# 54 C.I. ǀ 399 ǀ 20011498	C	*Aspergillus terreus*	27	8	20	8	8	30	5	8	7
# 56 C.I. ǀ 401 ǀ 20011967	C	*Aspergillus terreus*	13	35	19	4	13	15	9	9	7
# 57 C.I. ǀ 402 ǀ 20013386	C	*Aspergillus terreus*	27	8	20	8	8	33	5	8	7
# 58 C.I. ǀ 403 ǀ 20014048	C	*Aspergillus terreus*	27	8	20	8	8	30	5	8	7
# 61 C.I. ǀ 407 ǀ 20027127	C	*Aspergillus terreus*	9	12	45	12	13	-2	8	10	5
# 61a C.I. ǀ 407 ǀ 20027127	C	*Aspergillus terreus*	9	12	45	12	13	-2	8	10	5
# 62 C.I. ǀ 20006622	C	*Aspergillus terreus*	20	26	20	4	6	-2	5	5	14
# 63 C.I. ǀ 20034226	C	*Aspergillus terreus*	27	8	20	8	8	32	5	8	7
# 64 C.I. ǀ 20041068	C	*Aspergillus terreus*	5	12	42	5	17	-2	8	13	8
# 65 C.I. ǀ 20042006	C	*Aspergillus terreus*	27	8	20	8	8	31	5	8	7
# 66 C.I. ǀ 20055189	C	*Aspergillus terreus*	11	26	43	4	9	-2	5	7	13
# 67 C.I. ǀ 20055742	C	*Aspergillus terreus*	27	8	20	8	8	32	5	8	7
# 68 C.I. ǀ 20062392	C	*Aspergillus terreus*	20	26	20	4	6	-2	5	5	14

Supplementary Table S2

	Environmental isolates	CAS (1)	CAS (2)	AmB (1)	AmB (2)	VRC (1)	VRC (2)	POS (1)	POS (2)
**# 1**	11.06.2019-I-S-51	0,5	0,5	2	2	0,5	0,5	0,5	0,25
**# 2**	17.06.2019-I-S-33	0,25	0,25	2	2	0,5	0,5	0,25	0,5
**# 3**	17.06.2019-I-S-33	0,25	0,25	2	2	0,5	0,5	0,5	0,5
**# 4**	02.07.2019-KB-S-23	0,25	0,25	2	2	0,5	0,5	0,5	0,5
**# 5**	08.07.2019-I-S-1	0,25	0,5	4	4	0,5	0,5	0,5	0,5
**# 6**	08.07.2019-I-S-1	0,25	0,25	2	2	0,5	0,5	0,25	0,25
**# 7**	09.07.2019-I-P-10	0,25	0,25	2	2	0,5	0,5	0,5	0,5
**# 8**	11.07.2019-IL-S-41	0,25	0,25	2	2	0,5	0,5	0,5	0,5
**# 9**	11.07.2019-IL-S-41	0,25	0,25	2	2	0,5	0,5	0,5	0,5
**# 10**	17.07.2019-I-P-21	0,25	0,25	2	2	0,5	0,5	0,5	0,5
**# 11**	22.07.2019-KU-S-29	0,25	0,25	4	4	0,5	0,5	0,5	0,5
**# 12**	22.07.2019-KU-S-30	0,5	0,5	4	4	0,5	0,5	0,5	0,5
**# 13**	22.07.2019-KU-S-32	0,25	0,25	2	2	0,5	0,5	0,5	0,5
**# 14**	22.07.2019-KU-S-32	0,25	0,25	2	2	0,5	0,5	0,5	0,25
**# 15**	22.07.2019-KU-S-32	0,25	0,25	2	2	0,5	0,5	0,25	0,5
**# 16**	22.07.2019-KU-S-32	0,25	0,25	2	2	0,5	0,5	0,5	0,5
**# 17**	22.07.2019-KU-S-32	0,5	0,25	2	2	0,5	0,5	0,5	0,5
**# 18**	22.07.2019-KU-S-32	0,25	0,25	2	2	0,5	0,5	0,5	0,5
**# 19**	24.07.2019-KB-S-11	0,25	0,25	1	1	0,5	0,5	0,5	0,5
**# 20**	24.07.2019-KB-S-22	0,25	0,25	4	4	0,5	0,5	0,5	0,5
**# 21**	24.07.2019-KB-S-57	0,5	0,5	8	8	0,5	0,5	0,5	0,5
**# 22**	24.07.2019-KB-S-64	0,25	0,25	2	2	0,5	0,5	0,5	0,5
**# 23**	24.07.2019-KB-P-72	0,25	0,25	4	4	0,5	0,5	0,5	0,5
**# 24**	24.07.2019-KB-P-72	0,25	0,25	4	4	0,5	0,5	0,5	0,5
**# 25**	24.07.2019-KB-S-85	0,5	0,5	4	4	1	1	0,5	0,5
**# 26**	24.07.2019-KB-S-86	0,25	0,25	1	1	0,5	0,5	0,5	0,5

**# 27**	29.07.2019-I-S-2	0,5	0,5	4	4	0,5	0,5	0,5	0,5
**# 28**	29.07.2019-I-S-2	0,25	0,25	1	1	0,5	0,5	0,5	0,5
**# 29**	29.07.2019-I-S-2	0,5	0,5	2	2	0,5	0,5	0,5	0,5
**# 30**	29.07.2019-I-S-2	0,5	0,5	1	1	0,5	0,5	0,5	0,5
**# 32**	30.07.2019-KU-S-11	0,25	0,25	2	2	1	1	0,5	0,5
**# 33**	30.07.2019-KU-S-11	0,5	0,5	4	4	1	1	0,5	0,5
**# 34**	30.07.2019-KU-S-11	0,5	0,5	4	4	1	1	0,5	0,5
**# 36**	30.07.2019-KU-S-11	0,5	0,5	4	4	1	1	0,5	0,5
**# 37**	30.07.2019-KU-S-11	0,5	0,5	4	4	1	1	0,5	0,5
**# 38**	30.07.2019-KU-S-11	0,5	0,5	4	4	1	1	0,5	0,5
**# 39**	30.07.2019-KU-S-11	0,5	0,5	4	4	1	1	0,5	0,5
**# 40**	30.07.2019-KU-S-18	0,25	0,25	1	1	0,5	0,5	0,5	0,5
**# 41**	30.07.2019-KU-S-18	0,25	0,25	2	2	0,5	0,5	0,25	0,25
**# 42**	30.07.2019-KU-S-18	0,25	0,25	2	2	0,5	0,5	0,25	0,25
**# 43**	30.07.2019-KU-S-18	0,25	0,25	2	2	0,5	0,5	0,25	0,25
**# 44**	30.07.2019-KU-S-21	0,25	0,25	2	2	0,5	0,5	0,25	0,25
**# 45**	30.07.2019-KU-S-21	0,25	0,25	2	2	0,5	0,5	0,25	0,25
**# 46**	30.07.2019-KU-S-21	0,25	0,25	2	2	0,5	0,5	0,25	0,25
**# 47**	30.07.2019-SZ-S-45	0,25	0,25	2	2	0,5	0,5	0,25	0,25
**# 48**	30.07.2019-SZ-S-45	0,25	0,25	4	4	0,5	0,5	0,25	0,25
**# 49**	30.07.2019-KU-DM-101	0,25	0,25	2	2	0,5	0,5	0,25	0,25
**# 50**	01.08.2019-IL-S-7	0,125	0,125	8	8	0,5	0,5	0,25	0,25
**# 51**	01.08.2019-IL-S-7	0,25	0,25	4	2	0,5	0,5	0,25	0,25
**# 52**	07.08.2019-RE-S-21	0,25	0,25	2	2	0,5	0,5	0,25	0,25
**# 53**	07.08.2019-RE-S-35	0,25	0,25	8	8	0,5	0,5	0,25	0,25
**# 54**	07.08.2019-RE-S-52	0,25	0,25	8	8	0,5	0,5	0,25	0,25
**# 55**	07.08.2019-RE-A-81	0,25	0,25	2	2	0,5	0,5	0,125	0,25
**# 56**	12.08.2019-IL-S-27	0,25	0,25	2	2	0,5	0,5	0,25	0,25
**# 57**	20.08.2019-IL-S-11	0,25	0,25	4	4	0,5	0,5	0,25	0,25
**# 58**	22.08.2019-LA-S-55	0,5	0,5	4	4	0,5	0,5	0,25	0,25

**# 59**	22.08.2019-LA-S-62	0,25	0,25	2	2	0,5	0,5	0,25	0,25
**# 60**	27.08.2019-IL-S-5	0,25	0,25	2	2	0,5	0,5	0,25	0,25
**# 61**	27.08.2019-IL-S-22	0,25	0,25	2	2	0,5	0,5	0,25	0,25
**# 62**	27.08.2019-IL-A-25	0,25	0,25	4	4	1	1	0,25	0,25
**# 63**	27.08.2019-IL-S-28	0,25	0,25	2	2	0,5	0,5	0,25	0,25
**# 64**	27.08.2019-IL-S-54	0,25	0,25	4	4	0,5	0,5	0,25	0,25
**# 65**	27.08.2019-IL-S-57	0,25	0,25	2	2	0,5	0,5	0,125	0,25
**# 66**	27.08.2019-LZ-S-67	0,25	0,25	2	2	0,5	0,5	0,25	0,25
**# 67**	27.08.2019-KB-S/P-86	0,25	0,25	2	2	0,5	0,5	0,25	0,25
**# 68**	03.09.2019-LA-S-14	0,25	0,25	2	2	0,5	0,5	0,25	0,25
**# 69**	03.09.2019-SZ-S-22	0,25	0,25	1	0,5	0,5	0,5	0,25	0,25
**# 70**	03.09.2019-SZ-S-22	0,25	0,25	1	1	0,5	0,5	0,25	0,25
**# 71**	03.09.2019-SZ-S-22	0,25	0,25	4	4	1	1	0,5	0,5
**# 72**	03.09.2019-SZ-S-26	0,25	0,25	2	2	0,5	0,5	0,25	0,25
**# 73**	03.09.2019-SZ-S-29	0,25	0,25	4	4	0,5	0,5	0,5	0,5
**# 74**	03.09.2019-SZ-S-34	0,5	0,25	2	2	0,5	0,5	0,25	0,25
**# 75**	03.09.2019-SZ-S-37	0,25	0,25	4	4	1	1	0,25	0,25
**# 76**	03.09.2019-SZ-S-37	0,25	0,25	4	4	0,5	0,5	0,25	0,25
**# 77**	03.09.2019-SZ-S-43	0,25	0,25	4	4	0,5	0,5	0,25	0,25
**# 78**	03.09.2019-SZ-S-43	0,25	0,25	4	4	0,5	0,5	0,25	0,25
**# 79**	03.09.2019-SZ-S-44	0,25	0,25	8	8	1	1	0,25	0,25
**# 80**	03.09.2019-SZ-S-47	0,25	0,25	2	2	0,5	0,5	0,25	0,25
**# 81**	03.09.2019-SZ-S-47	0,25	0,25	2	2	0,5	0,5	0,25	0,25
**# 82**	03.09.2019-SZ-S-47	0,25	0,25	2	2	0,5	0,5	0,25	0,25
**# 83**	03.09.2019-SZ-S-47	0,25	0,25	2	2	0,5	0,5	0,25	0,25
**# 84**	03.09.2019-SZ-S-47	0,25	0,25	4	4	1	1	0,25	0,25
**# 85**	03.09.2019-SZ-P-59	0,25	0,25	2	2	0,5	0,5	0,25	0,25
**# 86**	03.09.2019-SZ-P-59	0,25	0,25	2	2	0,5	0,5	0,25	0,25
**# 87**	03.09.2019-SZ-P-65	0,25	0,25	2	2	0,5	0,5	0,25	0,25

**# 88**	03.09.2019-SZ-S-67	0,25	0,25	2	2	0,5	0,5	0,25	0,25
**# 89**	03.09.2019-SZ-S-67	0,25	0,25	4	4	0,5	0,5	0,25	0,25
**# 90**	03.09.2019-SZ-S-69	0,125	0,125	4	4	0,5	0,5	0,25	0,25
**# 91**	03.09.2019-SZ-S-71	0,25	0,25	4	4	0,5	0,5	0,25	0,25
**# 92**	03.09.2019-SZ-S-71	0,25	0,125	4	4	1	1	0,5	0,5
**# 93**	03.09.2019-SZ-S-73	0,25	0,25	4	4	0,5	0,5	0,25	0,25
**# 94**	17.06.2019-IL-A-AM-out	0,25	0,25	2	2	0,5	0,5	0,25	0,25
**# 95**	17.06.2019-IL-A-AM-out	0,25	0,25	2	2	0,5	0,5	0,25	0,25
**# 96**	17.06.2019-IL-A-MM-out	0,5	0,5	4	4	1	1	0,25	0,25
**# 97**	30.07.2019-SZ-S-45	0,25	0,25	2	2	0,5	0,5	0,25	0,25
**# 98**	10.09.2019-IM-S-14	0,25	0,25	2	2	0,5	0,5	0,25	0,25
**# 99**	10.09.2019-IM-S-40	0,25	0,25	4	4	0,5	0,5	0,25	0,25
**# 100**	19.09.2019-IL-S-13	0,25	0,125	2	2	0,5	0,5	0,25	0,25
**# 101**	19.09.2019-IL-S-13	0,25	0,25	2	2	0,5	0,5	0,25	0,25
**# 102**	19.09.2019-IL-S-13	0,25	0,25	4	4	0,5	0,5	0,5	0,5
**# 103**	19.09.2019-IL-S-13	0,25	0,25	4	4	0,5	0,5	0,5	0,5
**# 104**	19.09.2019-IL-S/DM-29	0,5	0,5	2	2	1	1	0,25	0,25
**# 106**	25.10.2019-KU-S-12	0,25	0,25	4	4	1	1	0,5	0,5
**# 107**	25.10.2019-KU-S-12	0,25	0,25	2	2	0,5	0,5	0,25	0,25
**# 108**	25.10.2019-KU-S-12	0,25	0,25	2	2	0,5	0,5	0,25	0,25
**# 109**	25.10.2019-KU-S-12	0,25	0,25	4	4	0,5	0,5	0,5	0,25
**# 111**	25.10.2019-KU-S-17	0,5	0,25	2	2	0,5	0,5	0,25	0,25
**# 112**	25.10.2019-KU-S-21	0,25	0,25	2	2	0,5	0,5	0,25	0,25
**# 113**	25.10.2019-KU-S-24	0,25	0,25	2	2	0,5	0,5	0,25	0,25
**# 114**	25.10.2019-KU-S-30	0,25	0,25	2	2	0,5	0,5	0,25	0,25
**# 115**	25.10.2019-KU-P-31	0,25	0,5	2	2	0,5	0,5	0,25	0,25
**# 116**	25.10.2019-KU-A-40	0,5	0,25	4	4	1	1	0,25	0,25
**# 117**	25.10.2019-KU-A-51	0,25	0,25	4	4	0,5	0,5	0,25	0,25

**# 118**	19.01.20 KB-A-1	0,25	0,25	4	4	1	1	0,25	0,5
**# 119**	19.0120 KB-A-2	0,25	0,25	2	2	0,5	0,5	0,25	0,25
**# 120**	19.01.20 KB-A-1	0,5	0,25	4	4	0,5	0,5	0,5	0,5
**# 121**	19.01.20 KB-A-2	0,5	0,25	4	4	0,5	0,5	0,5	0,5
**# 121a**	19.01.20 KB-A-2	0,5	0,25	4	4	0,5	0,5	0,5	0,5
**# 122**	17.01.2020-SZ-S-41	0,25	0,25	2	2	0,5	0,5	0,5	0,25
**# 123**	17.01.2020-SZ-S-41	0,25	0,25	2	2	0,5	0,5	0,5	0,25
**# 124**	17.01.2020-SZ-S-41	0,25	0,25	2	2	0,5	0,5	0,5	0,25
**# 125**	17.01.2020-SZ-S-55	0,25	0,25	2	2	0,5	0,5	0,25	0,25
**# 126**	17.01.2020-KU-S-66	0,25	0,25	2	2	0,5	0,5	0,25	0,25
**# 127**	17.01.2020-KU-A-68	0,25	0,25	4	4	0,5	0,5	0,25	0,25
**# 128**	17.01.2020-KU-A-74	0,25	0,25	1	1	0,25	0,25	0,25	0,25
**# 129**	17.01.2020-KU-A-77	0,25	0,25	4	4	0,5	0,5	0,25	0,25
**# 130**	17.01.2020-KU-A-83	0,25	0,25	4	4	0,5	0,5	0,25	0,25
**# 131**	17.01.2020-KU-A-87	0,25	0,25	2	2	0,5	0,5	0,25	0,25
**# 132**	17.01.2020-KU-A-92	0,5	0,5	4	4	0,5	0,5	0,25	0,25
**# 133**	17.01.2020-KU-S-94	0,25	0,25	4	4	0,5	0,5	0,125	0,125
**# 134**	17.01.2020-KU-S-106	0,25	0,25	4	4	0,5	0,5	0,25	0,25
**# 135**	23.01.2020-SZ-S-1	0,25	0,25	4	4	0,5	0,5	0,25	0,25
**# 136**	23.01.2020-SZ-S-2	0,25	0,25	2	2	0,5	0,5	0,25	0,25
**# 137**	23.01.2020-SZ-S-7	0,25	0,25	4	4	0,5	0,5	0,125	0,125
**# 138**	23.01.2020-SZ-S-8	0,25	0,25	4	4	0,5	0,5	0,25	0,25
**# 139**	23.01.2020-SZ-DM-9	0,25	0,25	4	4	1	1	0,25	0,25
**# 140**	23.01.2020-SZ-DM-12	0,25	0,25	4	4	0,5	0,5	0,125	0,125
**# 141**	23.01.2020-SZ-A-13	0,25	0,25	1	1	0,5	0,5	0,25	0,25
**# 142**	23.01.2020-SZ-S-14	0,25	0,25	1	1	0,5	0,5	0,25	0,25
**# 143**	23.01.2020-SZ-DM-16	0,25	0,25	8	8	0,5	0,5	0,25	0,25
**# 144**	23.01.2020-SZ-P-18	0,25	0,25	2	2	0,5	0,5	0,25	0,25

**# 145**	23.01.2020-SZ-P-19	0,25	0,25	1	1	0,5	0,5	0,25	0,25
**# 146**	23.01.2020-SZ-S-23	0,25	0,25	2	2	0,5	0,5	0,25	0,25
**# 147**	23.01.2020-SZ-DM-24	0,25	0,25	2	2	0,5	0,5	0,25	0,25
**# 148**	23.01.2020-SZ-DM-25	0,25	0,25	8	8	0,5	0,5	0,25	0,25
**# 149**	23.01.2020-SZ-A-26	0,25	0,25	2	2	0,5	0,5	0,25	0,25
**# 150**	23.01.2020-SZ-S-31	0,25	0,25	2	2	0,5	0,5	0,25	0,25
**# 151**	23.01.2020-SZ-A-33	0,25	0,25	2	2	0,5	0,5	0,25	0,25
**# 152**	23.01.2020-SZ-S-34	0,25	0,25	2	2	0,5	0,5	0,25	0,25
**# 153**	23.01.2020-SZ-P-36	0,5	0,5	4	4	0,5	0,5	0,125	0,125
**# 154**	23.01.2020-SZ-A-37	0,5	0,5	4	4	1	1	1	1
**# 155**	23.01.2020-SZ-S-41	0,25	0,25	4	4	0,5	0,5	0,25	0,25
**# 156**	23.01.2020-SZ-P-42	0,25	0,25	2	2	0,5	0,5	0,25	0,25
**# 157**	23.01.2020-SZ-A-46	0,25	0,25	4	4	0,5	0,5	0,5	0,25
**# 158**	23.01.2020-SZ-S-48	0,25	0,25	4	4	0,5	0,5	0,125	0,125
**# 159**	23.01.2020-SZ-S-54	0,25	0,25	4	4	0,5	0,5	0,125	0,125
**# 160**	23.01.2020-SZ-S-56	0,25	0,25	4	4	0,25	0,25	0,25	0,25
**# 161**	23.01.2020-SZ-S-62	0,25	0,25	4	4	0,5	0,5	0,25	0,25
**# 162**	23.01.2020-SZ-P-63	0,25	0,25	2	2	0,5	0,5	0,25	0,25
**# 163**	23.01.2020-SZ-DM-64	0,25	0,25	4	4	0,5	0,5	0,25	0,25
**# 164**	23.01.2020-SZ-DM-65	0,125	0,125	4	4	0,5	0,5	0,125	0,125
**# 165**	23.01.2020-SZ-S-66	0,25	0,25	2	2	0,5	0,5	0,125	0,125
**# 166**	23.01.2020-SZ-S-67	0,25	0,25	1	1	0,25	0,25	0,125	0,125
**# 167**	23.01.2020-SZ-P-68	0,25	0,25	2	2	0,25	0,25	0,125	0,125
**# 168**	23.01.2020-SZ-A-73	0,25	0,25	4	4	0,5	0,5	0,25	0,25
**# 169**	23.01.2020-SZ-P-76	0,25	0,25	8	8	0,5	0,5	0,125	0,125
**# 170**	23.01.2020-SZ-S-87	0,25	0,25	2	2	0,5	0,5	0,25	0,25
**# 171**	23.01.2020-SZ-S-93	0,25	0,25	4	4	0,5	0,5	0,25	0,25
**# 172**	30.01.2020-KU-P-12	0,25	0,25	4	4	0,5	0,5	0,25	0,25

**# 173**	30.01.2020-KU-S-19	0,25	0,25	2	2	0,5	0,5	0,25	0,25
**# 174**	30.01.2020-KU-S-31	0,25	0,25	2	2	0,5	0,5	0,25	0,25
**# 175**	30.01.2020-KU-DM-34	0,25	0,25	2	2	0,5	0,5	0,25	0,25
**# 176**	30.01.2020-KU-S-41	0,25	0,25	4	4	0,5	0,5	0,125	0,125
**# 177**	30.01.2020-KU-P-45	0,25	0,25	2	2	0,5	0,5	0,25	0,25
**# 178**	30.01.2020-SZ-DM/P-72	0,25	0,25	2	2	0,5	0,5	0,25	0,25
**# 179**	30.01.2020-SZ-P-75	0,25	0,25	2	2	0,5	0,5	0,25	0,25
**# 180**	06.02.2020-IL-A-8	0,25	0,25	2	2	0,5	0,5	0,25	0,25
**# 181**	06.02.2020-IM-S-57	0,5	0,5	2	2	0,5	0,5	0,5	0,5
**# 182**	06.02.2020-IM-S-62	0,25	0,25	2	2	0,5	0,5	0,25	0,25
**#183**	14.02.2020-IL-S-21	0,25	0,25	4	4	1	1	0,25	0,25
**#184**	14.02.2020-IL-DM-30	0,5	0,5	4	4	0,25	0,25	0,125	0,125
**#185**	14.02.2020-IL-S-72	0,25	0,25	2	2	0,125	0,125	0,25	0,25
**#186**	20.02.2020-I-S-43	0,25	0,25	2	2	0,25	0,25	0,25	0,25
**#187**	27.02.2020-I-W-4	0,25	0,25	2	2	0,25	0,25	0,25	0,25
**#188**	05.03.2020-KU-S-7	0,25	0,25	2	2	0,5	0,5	0,25	0,25
**#189**	05.03.2020-KU-S-15	0,25	0,25	4	4	0,5	0,5	0,25	0,25
**#190**	05.03.2020-KU-P-17	0,25	0,25	4	4	0,5	0,5	0,25	0,25
**#191**	05.03.2020-KU-DM-20	0,25	0,25	4	4	0,5	0,5	0,25	0,25
**#192**	05.03.2020-KU-S-24	0,25	0,25	2	2	0,5	0,5	0,25	0,25
**#193**	05.03.2020-KU-S-27	0,25	0,25	2	2	0,5	0,5	0,25	0,25
**#194**	05.03.2020-KU-S-35	0,25	0,25	2	2	0,5	0,5	0,25	0,25
**#195**	05.03.2020-KU-P-42	0,25	0,25	4	4	0,5	0,5	0,25	0,25
**#196**	05.03.2020-KU-S-43	0,25	0,25	2	2	0,5	0,5	0,25	0,25
**#197**	05.03.2020-KU-P-58	0,25	0,25	2	2	0,5	0,5	0,25	0,25
**#198**	05.03.2020-KU-S-59	0,25	0,25	2	2	0,5	0,5	0,25	0,25
**#199**	05.03.2020-KB-W-77	0,25	0,25	2	2	0,5	0,5	0,25	0,25
**#200**	05.03.2020-I-S-101	0,125	0,125	2	2	0,5	0,5	0,25	0,25

**#201**	11.03.2020-IL-DM-7	0,25	0,25	4	4	0,5	0,5	0,25	0,25
**#202**	11.03.2020-IL-S-29	0,5	0,5	1	1	0,5	0,5	0,5	0,5
**#203**	15.04.2020-I-S-18	1	1	4	4	0,25	0,25	0,125	0,125
**#204**	15.04.2020-I-S-20	0,25	0,25	2	2	0,5	0,5	0,125	0,125
**#205**	15.04.2020-I-S-74	0,25	0,25	4	4	0,5	0,5	0,25	0,25
**#206**	15.04.2020-KB-A-6	0,5	0,5	4	4	1	1	0,25	0,25
**#207**	15.04.2020-KB-S-15	0,25	0,25	2	2	0,5	0,5	0,25	0,25
**#208**	15.04.2020-KB-E-18	0,25	0,25	2	2	0,5	0,5	0,25	0,25
**#209**	15.04.2020-KB-S-25	0,25	0,25	4	4	0,5	0,5	0,25	0,25
**#210**	15.04.2020-KB-S-60	0,5	0,25	2	2	0,5	0,5	0,25	0,25
**#211**	15.04.2020-KB-S-65	0,25	0,25	8	8	1	1	0,5	0,5
**#212**	15.04.2020-KB-S-75	0,5	0,5	2	2	0,5	0,5	0,5	0,5
**#213**	15.04.2020-KB-S-83	0,5	0,5	2	2	0,5	0,5	0,25	0,25
**#214**	15.04.2020-KB-S-93	0,25	0,25	4	4	0,5	0,5	0,25	0,25
**#215**	15.04.2020-KB-S-94	0,25	0,25	1	1	0,5	0,5	0,25	0,25
**#216**	17.04.2020-I-A-39	0,25	0,25	1	1	0,5	0,5	0,25	0,125
**#217**	17.04.2020-I-S-58	0,25	0,25	4	4	0,5	0,5	0,0625	0,0625
**#218**	17.04.2020-I-S-87	0,5	0,5	4	4	0,5	0,5	0,25	0,25
**#219**	15.04.2020-KB-A-9	0,25	0,25	2	2	0,5	0,5	0,25	0,25
**#220**	20.04.2020-RE-S-34	0,25	0,25	1	1	0,5	0,5	0,25	0,25
**#221**	20.04.2020-RE-S-54	0,25	0,25	2	2	1	1	0,25	0,25
**#222**	20.04.2020-RE-S-94	0,25	0,5	2	2	1	1	0,25	0,25
**#223**	22.04.2020-I-S-6	0,5	0,5	0,5	0,5	0,25	0,25	0,25	0,25
**#224**	22.04.2020-I-S-11	0,25	0,25	4	4	0,5	0,5	0,25	0,25
**#225**	22.04.2020-I-S-34	0,25	0,25	2	2	0,5	0,5	0,25	0,25
**#226**	22.04.2020-I-S-49	0,25	0,25	2	2	0,5	0,5	0,25	0,25
**#227**	23.04.2020-IM-P-20	0,25	0,25	2	2	0,5	0,5	0,25	0,25
**#228**	23.04.2020-IM-DM-86	0,25	0,25	4	4	0,5	0,5	0,25	0,25
**#229**	29.04.2020-I-S-31	0,5	0,5	2	2	0,5	0,5	0,25	0,25
**#230**	29.04.2020-I-S-48	0,25	0,25	2	2	0,5	0,5	0,25	0,25

**#231**	20.04.2020-RE-S-84	0,25	0,25	4	4	0,5	0,5	0,25	0,25
**#232**	30.04.2020-IM-S-5	0,25	0,5	2	2	0,5	0,5	0,25	0,25
**#233**	30.04.2020-LA-P-14	0,5	0,5	2	2	0,5	0,5	0,25	0,25
**#234**	30.04.2020-LA-S-29	0,25	0,25	1	1	0,5	0,5	0,25	0,25
**#235**	30.04.2020-LA-S-32	0,5	0,5	2	2	0,5	0,5	0,25	0,25
**#236**	30.04.2020-LA-S-77	0,5	0,5	2	2	0,5	0,5	0,25	0,25
**#237**	08.05.2020-IL-S-26	0,25	0,25	4	4	0,5	0,5	0,25	0,25
**#238**	08.05.2020-IL-S-40	0,25	0,25	2	2	0,5	0,5	0,5	0,5
**#239**	08.05.2020-IL-S-53	0,25	0,25	4	4	1	1	0,25	0,25
**#240**	08.05.2020-IL-P-54	0,5	0,25	4	4	0,5	0,5	0,25	0,25
**#241**	08.05.2020-IL-P-54	0,25	0,25	2	2	0,5	0,5	0,25	0,25
**#242**	08.05.2020-IL-S-80	0,25	0,25	2	2	0,5	0,5	0,25	0,25
	**Clinical isolates**								
		**CAS (1)**	**CAS (2)**	**AmB (1)**	**AmB (2)**	**VRC (1)**	**VRC (2)**	**POS (1)**	**POS (2)**
**# 1 C.I.**	Clinical isolate	0,125	0,125	4	4	0,5	0,5	0,25	0,25
**# 2 C.I.**	Clinical isolate	0,25	0,25	4	4	0,5	0,5	0,5	0,25
**# 3 C.I.**	Clinical isolate	0,25	0,25	4	4	0,25	0,25	0,125	0,125
**# 4 C.I.**	Clinical isolate	0,125	0,125	4	4	0,5	0,5	0,125	0,25
**# 5 C.I.**	Clinical isolate	0,25	0,25	4	4	0,25	0,25	0,125	0,25
**# 7 C.I.**	Clinical isolate	0,25	0,25	4	4	0,5	0,5	0,063	0,063
**# 8 C.I.**	Clinical isolate	0,25	0,25	4	4	0,5	0,5	0,25	0,25
**# 9 C.I.**	Clinical isolate	0,5	0,25	4	4	2	2	0,25	0,25
**# 10 C.I.**	Clinical isolate	0,25	0,25	4	4	0,25	0,25	0,25	0,25
**# 11 C.I.**	Clinical isolate	0,25	0,25	4	4	0,25	0,25	0,5	0,25
**# 12 C.I.**	Clinical isolate	0,25	0,25	4	4	0,25	0,25	0,125	0,125
**# 13 C.I.**	Clinical isolate	0,5	0,5	8	8	0,5	0,5	0,25	0,25
**# 14 C.I.**	Clinical isolate	0,25	0,25	4	4	0,25	0,25	0,25	0,25
**# 15 C.I.**	Clinical isolate	0,125	0,125	2	2	0,5	0,5	0,25	0,25

**# 16 C.I.**	Clinical isolate	0,25	0,25	1	1	0,25	0,25	0,25	0,25
**# 17 C.I.**	Clinical isolate	0,25	0,25	8	8	0,5	0,5	0,5	0,25
**# 18 C.I.**	Clinical isolate	0,25	0,25	4	4	0,5	0,5	0,25	0,25
**# 20 C.I.**	Clinical isolate	0,25	0,25	4	4	0,5	0,5	0,125	0,125
**# 21 C.I.**	Clinical isolate	0,25	0,25	4	4	0,5	0,5	0,25	0,25
**# 22 C.I.**	Clinical isolate	0,25	0,25	4	4	0,25	0,25	0,25	0,25
**# 23 C.I.**	Clinical isolate	0,25	0,25	4	4	0,25	0,25	0,25	0,25
**# 25 C.I.**	Clinical isolate	0,25	0,25	4	4	0,5	0,5	0,25	0,25
**# 26 C.I.**	Clinical isolate	0,25	0,25	>16	>16	1	1	0,25	0,25
**# 27 C.I.**	Clinical isolate	0,25	0,25	8	8	0,5	0,5	0,25	0,25
**# 28 C.I.**	Clinical isolate	0,5	0,5	8	8	0,5	0,5	0,125	0,125
**# 30 C.I.**	Clinical isolate	0,25	0,25	4	4	0,5	0,5	0,25	0,25
**# 31 C.I.**	Clinical isolate	0,25	0,25	2	2	0,5	0,5	0,5	0,25
**# 33 C.I.**	Clinical isolate	0,5	0,5	16	16	0,5	0,5	0,25	0,125
**# 34 C.I.**	Clinical isolate	0,25	0,25	4	4	0,25	0,25	0,25	0,25
**# 35 C.I.**	Clinical isolate	0,25	0,25	4	4	0,5	0,5	0,25	0,25
**# 36 C.I.**	Clinical isolate	0,25	0,25	4	4	0,5	0,5	0,25	0,25
**# 38 C.I.**	Clinical isolate	0,5	0,5	4	4	0,25	0,25	0,25	0,25
**# 39 C.I.**	Clinical isolate	0,25	0,25	4	4	0,5	0,5	0,25	0,25
**# 40 C.I.**	Clinical isolate	0,25	0,25	8	8	0,5	0,5	0,25	0,25
**# 43 C.I.**	Clinical isolate	0,25	0,25	4	4	1	1	0,125	0,125
**# 44 C.I.**	Clinical isolate	0,25	0,25	4	4	0,5	0,5	0,25	0,25
**# 45 C.I.**	Clinical isolate	0,25	0,25	8	8	0,5	0,5	0,25	0,25
**# 46 C.I.**	Clinical isolate	0,25	0,25	4	4	4	4	0,125	0,125
**# 51 C.I.**	Clinical isolate	0,25	0,25	4	4	0,5	0,5	0,25	0,25
**# 53 C.I.**	Clinical isolate	0,25	0,5	4	4	1	1	0,25	0,5
**# 54 C.I.**	Clinical isolate	0,25	0,25	4	4	0,5	0,5	0,25	0,25
**# 56 C.I.**	Clinical isolate	0,25	0,25	8	8	0,5	0,5	0,25	0,25

**# 57 C.I.**	Clinical isolate	0,5	0,5	2	2	0,5	0,5	0,25	0,25
**# 58 C.I.**	Clinical isolate	0,25	0,25	4	4	0,5	0,5	0,25	0,25
**# 61 C.I.**	Clinical isolate	0,25	0,25	2	2	1	1	0,25	0,25
**# 61a C.I.**	Clinical isolate	0,25	0,25	2	2	1	1	0,25	0,25
**# 62 C.I.**	Clinical isolate	0,25	0,25	2	2	0,25	0,25	0,125	0,125
**# 63 C.I.**	Clinical isolate	0,25	0,25	2	2	0,25	0,25	0,125	0,125
**# 64 C.I**	Clinical isolate	0,25	0,25	4	4	4	4	0,125	0,125
**# 65 C.I**	Clinical isolate	0,25	0,25	>16	>16	1	1	0,25	0,25
**# 66 C.I**	Clinical isolate	0,25	0,25	2	2	0,5	0,5	0,25	0,25
**# 67 C.I**	Clinical isolate	0,25	0,25	2	2	0,25	0,25	0,125	0,125
**# 68 C.I**	Clinical isolate	0,25	0,25	4	4	0,25	0,25	0,25	0,25
